# Redox Isomerism in the S_3_ State of the Oxygen‐Evolving Complex Resolved by Coupled Cluster Theory

**DOI:** 10.1002/chem.202101567

**Published:** 2021-08-06

**Authors:** Maria Drosou, Dimitrios A. Pantazis

**Affiliations:** ^1^ Inorganic Chemistry Laboratory National and Kapodistrian University of Athens Panepistimiopolis Zografou 15771 Greece; ^2^ Max-Planck-Institut für Kohlenforschung Kaiser-Wilhelm-Platz 1 45470 Mülheim an der Ruhr Germany

**Keywords:** ab initio calculations, coupled cluster theory, photosynthesis, spin states, water oxidation

## Abstract

The electronic and geometric structures of the water‐oxidizing complex of photosystem II in the steps of the catalytic cycle that precede dioxygen evolution remain hotly debated. Recent structural and spectroscopic investigations support contradictory redox formulations for the active‐site Mn_4_CaO_*x*_ cofactor in the final metastable S_3_ state. These range from the widely accepted Mn^IV^_4_ oxo‐hydroxo model, which presumes that O−O bond formation occurs in the ultimate transient intermediate (S_4_) of the catalytic cycle, to a Mn^III^_2_Mn^IV^_2_ peroxo model representative of the contrasting “early‐onset” O−O bond formation hypothesis. Density functional theory energetics of suggested S_3_ redox isomers are inconclusive because of extreme functional dependence. Here, we use the power of the domain‐based local pair natural orbital approach to coupled cluster theory, DLPNO‐CCSD(T), to present the first correlated wave function theory calculations of relative stabilities for distinct redox‐isomeric forms of the S_3_ state. Our results enabled us to evaluate conflicting models for the S_3_ state of the oxygen‐evolving complex (OEC) and to quantify the accuracy of lower‐level theoretical approaches. Our assessment of the relevance of distinct redox‐isomeric forms for the mechanism of biological water oxidation strongly disfavors the scenario of early‐onset O−O formation advanced by literal interpretations of certain crystallographic models. This work serves as a case study in the application of modern coupled cluster implementations to redox isomerism problems in oligonuclear transition metal systems.

## Graphical abstract

**The domain‐based** local pair natural orbital–coupled cluster approach, DLPNO‐CCSD(T), was employed to obtain reliable relative energetics for distinct redox‐isomeric forms of the Mn_4_Ca cofactor of the oxygen‐evolving complex of photosystem II in its final metastable S_3_ state. It is shown that the Mn^IV^_4_ oxo‐hydroxo model is significantly more stable than the Mn^III^_2_Mn^IV^_2_ peroxo model, thus disfavoring the scenario of early‐onset O−O bond formation in biological water oxidation.

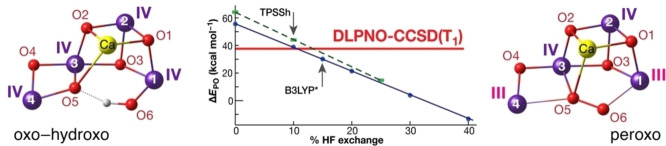

## Introduction

**Figure 1:**
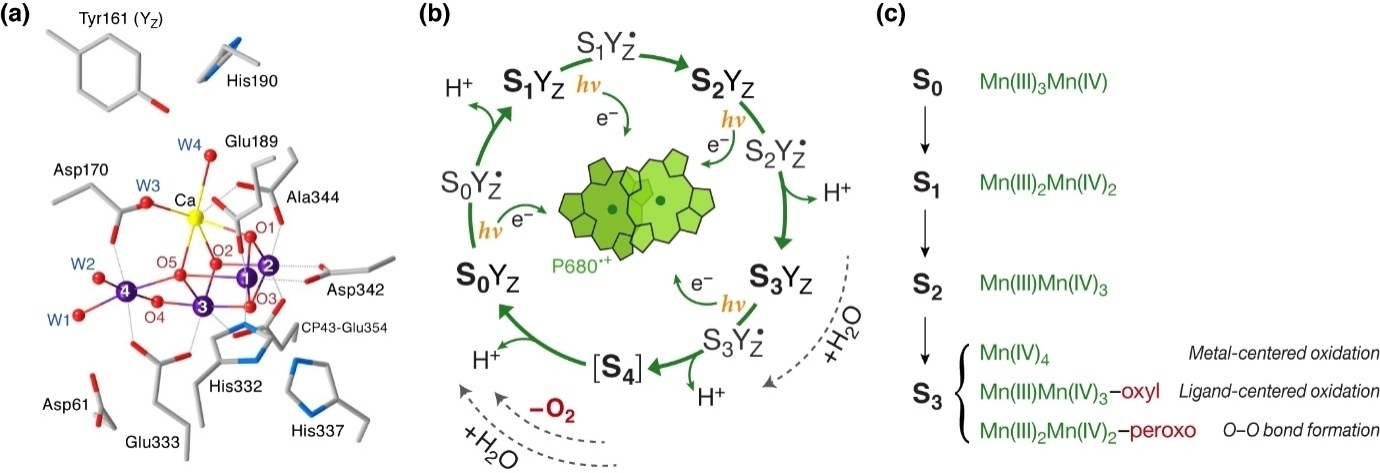
a) The catalytically active site of the OEC in the crystallographically best‐resolved S_1_ state. The Mn_4_CaO_5_ cluster is ligated by amino acid residues and water‐derived ligands. In the most common protonation state assignment W1, W3, and W4 are H_2_O and W2 is either H_2_O or OH. The redox‐active Tyr161 (known as Y_Z_) mediates electron transfer between the OEC and the charge‐separation site of photosystem II. b) Catalytic cycle of S_*i*_ states involved in the four‐electron oxidation of water to molecular oxygen by the OEC of photosystem II. The intermediate metalloradical states formed upon oxidation of the Y_Z_ residue are also indicated. The phenomenological observation of dioxygen evolution every four light flashes does not constrain the chemical nature of S_*i*_ intermediates. c) The assignment of Mn oxidation states is generally accepted for states S_0_, S_1_, and S_2_ states, that is, metal‐based storage of oxidizing equivalents in the first two transitions. However, it remains contested for the S_3_ state, where currently discussed suggestions include the limiting cases of continuing metal‐based oxidation to an all‐Mn^IV^ species, as well as the contrasting scenario of early‐onset O−O (peroxo) bond formation.

The active site of the oxygen‐evolving complex (OEC) of photosystem II contains a cluster of four Mn and one Ca ions linked by oxo bridges, Mn_4_CaO_*x*_ (Figure 1a).^[1]^ The OEC achieves the four‐electron oxidation of water to molecular dioxygen through a light‐driven catalytic cycle that consists of five distinct states S_*i*_, where *i*=0–4 denotes the number of extracted electrons (Figure 1b). The mechanism of O−O bond formation has been intensely researched, but remains contested.^[2]^ A major uncertainty centers on the precise composition of the S_3_ state,[^2a^, ^2b^, ^2c^] the last observable intermediate of the cycle prior to the rapid and unresolved oxygen‐evolving S_3_→[S_4_]→S_0_ transition. Different experimental and theoretical studies have provided support for fundamentally distinct geometric and electronic structures. No single structural model can fully describe the S_3_ state because of its heterogeneous nature that may arise in part from different water binding situations^[3]^ (note that S_3_ heterogeneity remains unresolved by crystallographic studies^[4]^ yet is revealed by magnetic resonance spectroscopy[^3a^, ^5^]). Nevertheless, a clear distinction can be made between two major types of model currently considered: i) those that assume continuous *storage* of oxidizing equivalents at least up to and including the S_3_ state, therefore precluding O−O bond formation prior to the final oxidation step (S_3_→[S_4_]) of the catalytic cycle, and ii) those that assume early‐onset formation of the O−O bond – of unspecified order – already in the S_3_ state, therefore restricting the genuine storage part of the cycle to the two lowest S_*i*_→S_*i*+1_ transitions. The implications for the mechanism of water oxidation are enormous, because the two possibilities lead to profoundly different scenarios in terms of the distinct redox transformations implicated in overall water oxidation, the number of electrons and nature of intermediates involved in each elementary step, and the partitioning of the S_*i*_‐state cycle into storage versus catalytic transitions.

**Figure 2:**
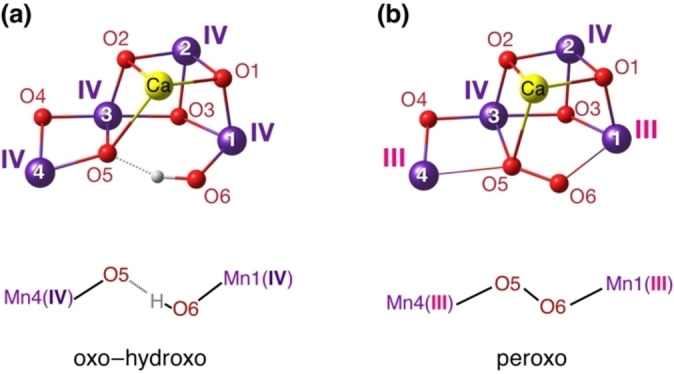
a) Example of an oxo‐hydroxo model for the S_3_ state (only core atoms shown for clarity), where all cluster manganese ions are Mn^IV^, following metal‐based oxidation of the S_2_‐state Mn^III^Mn^IV^_3_ cluster and ligation of an additional terminal water‐derived ligand that appears as O6H. This model has strong and direct support from spectroscopy. b) Example of a peroxo model for the S_3_ state, where an O5−O6 bond is already formed, and the Mn ions have oxidation states Mn^III^_2_Mn^IV^_2_, effectively identical to the dark‐stable S_1_ state. This model appears structurally consistent with certain XFEL crystallographic models,[^4b^, ^4c^, ^4d^] but is harder to reconcile with the electronic structure of the cluster implied by spectroscopic observations.^[2c]^

A widely accepted representative of the first type defined above is the so‐called “oxo‐hydroxo” model (Figure 2a). This is considered to be the result of successive metal‐centered oxidations in the S_0_→S_1_, S_1_→S_2_, and S_2_→S_3_ transitions that continuously raise the metal oxidation states from Mn^III^_3_Mn^IV^ in S_0_ to Mn^IV^_4_ in S_3_. The removal of three electrons from S_0_ to S_3_ is accompanied by alternate removal of two protons in the S_0_→S_1_ and S_2_→S_3_ transitions.^[6]^ Moreover, a water molecule is thought to bind in the S_3_ state,^[7]^ appearing directly or through internal rearrangements[^2b^, ^2c^, ^3b^, ^8^] as a new, possibly deprotonated, terminal ligand (O6 in Figure 2). The distance between the two internal O atoms (O5 and O6) is expected to be in the range ∼2.4–2.6 Å, typical of hydrogen bonding. This model of the S_3_ state is well supported by spectroscopy[^5a^, ^9^] and, in a broad sense, by structural studies that document an increase in the number of oxygen ligands compared to lower S_*i*_ states.[^4b^, ^4c^, ^4d^] Subsequent oxidation and deprotonation is suggested to create a reactive Mn^IV^‐oxyl radical^[10]^ in the [S_4_] state that initiates O5−O6 bond formation through oxo‐oxyl coupling.^[11]^

The above stand in stark contrast to the Mn^III^_2_Mn^IV^_2_ peroxo model depicted in Figure 2b, which assumes that water oxidation and O−O bond formation is well underway already in the S_3_ state.^[12]^ This scenario suggests a much earlier initiation of the genuine catalytic phase of the cycle, with the last oxidative step past the S_3_ state merely *completing* dioxygen formation rather than triggering it. The peroxo model is weakly correlated with spectroscopic observations,[^2c^, ^20^] which are instead consistent with Mn‐centered oxidations up to and including the S_2_→S_3_ transition rather than with reversal to the dark‐stable (resting) S_1_ Mn oxidation states. However, the peroxo model, or variants thereof with different fractional O−O bond orders,[^13^, ^20^] are consistent with face‐value interpretations of recent crystallographic models based on studies that utilized X‐ray free‐electron laser (XFEL) pulses. Despite considerable experimental uncertainties and non‐negligible differences among XFEL crystallographic models of the S_3_ state that do support the presence of an additional O ligand (O6),[^4b^, ^4c^, ^4d^] they all depict very short O5−O6 distances (in one case down to 1.45 Å,^[4b]^ typical of a peroxo bond) and accompanying Mn coordination geometries resembling Mn^III^ rather than Mn^IV^ ions.^[2c]^

Computational studies have been useful in demonstrating that both alternative redox formulations for the S_3_ state are realistic and that extensive redox equilibria can be formulated that involve the two models shown above.[^13^, ^14^] The proposed redox equilibria even extend to Mn^III^_3_Mn^IV^ superoxo[^13^, ^14^] (equivalent to the metal valence level of the S_0_ state), which is however incompatible with all structural and spectroscopic evidence on the S_3_ state and thus is not considered a realistic option. Inescapably, theoretical studies that focus on either structural or spectroscopic properties of computational models cannot be conclusive on the agreement of different redox forms with experiment, simply because at this point in time the spectroscopic data that demonstrate S_2_→S_3_ metal‐based oxidation and the XFEL structural interpretations that imply S_2_→S_3_ metal‐based reduction are inherently irreconcilable. Reliable computed energetics for different redox isomers can thus be a crucial independent piece of information for evaluating the different possibilities. However, the different redox isomers have different numbers of total *unpaired* electrons, a highly unfavorable scenario for density functional theory (DFT), for which the relative energies of isoelectronic species with different numbers of unpaired electrons depend strongly on the admixture of exact (Hartree−Fock) exchange.^[15]^ In broad terms, electron pair repulsion is stronger for intra‐orbital than inter‐orbital paired electrons and as the number of paired electrons is greater in states of lower than higher total spin, increased dynamic correlation is expected in lower‐spin states. For this reason, Hartree−Fock systematically overstabilizes high‐spin states. By contrast, pure DFT functionals tend to overstabilize the low‐spin states, because they tend to overestimate nondynamic electron correlation, especially in systems where the low‐spin state has a more delocalized charge distribution, such as when spin crossover involves electron promotion from a metal non‐bonding to a metal−ligand antibonding orbital.^[16]^ Therefore balanced retrieval of correlation energy across all states/configurations is critical for computing accurate relative energies. The situation with the S_3_ isomers is reminiscent of similar problems in quantum (bio)inorganic chemistry involving metal–oxygen adducts^[17]^ or indeed of the classical dichotomy between Cu^II,II^‐bis‐μ‐oxo and Cu^I,I^‐peroxo formulations of the Cu_2_O_2_ core,^[18]^ albeit magnified here by the greater number of open‐shell sites and total unpaired electrons.

Among wavefunction‐based electron correlation approaches, the coupled cluster with singles, doubles and perturbative triples CCSD(T) method is considered the “gold standard” of quantum chemistry due to its excellent accuracy, provided that the system can be adequately described by a single determinant.^[19]^ The value of CCSD(T) for studying spin‐state energetics of transition metal systems is amply documented,^[20]^ but OEC models of realistic size are way beyond the reach of all conventional coupled cluster implementations. The domain‐based local pair natural orbital approach, DLPNO‐CCSD(T),^[21]^ offers a highly efficient way of extending the applicability of CCSD(T) to large systems. The central idea is to use localized internal orbitals and projected atomic orbitals (PAOs) to describe the virtual space, in order to compress the information content of the CC wavefunction such that only a small set of amplitudes contains all essential information. The approach has been shown to have drastically reduced cost and near‐linear scaling with respect to system size while achieving performance nearly equivalent to the canonical CCSD(T) counterpart.^[22]^ Despite exploratory applications in isomerization studies of highly truncated models of the OEC for the early catalytic states,^[23]^ no study of redox energetics in the crucial S_3_ state has been attempted so far. Here we leverage the ability of DLPNO‐CCSD(T) to provide reference values for spin state energetics^[24]^ in order to achieve a reliable estimation of the energy difference between S_3_ Mn^IV^_4_ oxo‐hydroxo and the Mn^III^_2_Mn^IV^_2_ peroxo structures. The results enable us to provide a realistic and reliable energy profile of these redox isomers and to assess their relevance for the interpretation of experimental observations and for the mechanism of water oxidation. In addition, our results enable a rigorous assessment of hybrid density functional theory in order to identify the optimal approach for the treatment of significantly expanded models in analogous problems of redox‐isomerism energetics.

## Computational Methods

**OEC models**: The initial structure for the OEC models was taken from the latest crystallographic model of PSII reported by Suga et al. (PDB ID: 6JLL, monomer A).^[4c]^ The model used in the initial coupled cluster calculations consists of 126 atoms (Figure S1 in the Supporting Information), including the amino acid residues coordinated on the first coordination sphere of the Mn_4_CaO_5_ cofactor: His332, His337, Glu333, Glu189, Asp170, Asp342 and Ala344 from protein chain D1 and CP43‐Glu354, plus two H_2_O molecules (W1 as an H_2_O and W2 in its hydroxo or aquo form for the oxo‐hydroxo and peroxo forms, respectively) on the terminal Mn4 ion, as well as two H_2_O molecules on the Ca^2+^ ion. The second coordination sphere residues His337, Tyr161, which was replaced with a methanol molecule, and two water molecules hydrogen bonded to W3 plus a water molecule hydrogen bonded to O1, were considered essential to be included in the model, in order to preserve important structural features modulated by hydrogen bond networks. As emphasized before, models that simply omit Tyr161 without exactly preserving the associated hydrogen bonding network are not valid representations of the OEC. The extended OEC models consist of 325 atoms and, in addition to the aforementioned amino‐acid residues, they include: Asp61, Tyr161, Gln165, Ser169, Asn181, Val185, Asn298, His190, Leu343 from protein chain D1, Lys317 from protein chain D2 and CP43‐Arg357 (Figure S1). During geometry optimization, backbone constraints from the crystallographic coordinates were applied to the α‐carbon atoms of peptide bonds and to hydrogen atoms that replaced peripheral carbon atoms in order to simulate steric effects imposed on the OEC by the protein matrix (Figure S2). The oxo‐hydroxo and peroxo structures have the same number of atoms and electrons as well as the same constrained atoms during geometry optimization so that direct energy comparisons are meaningful.

**Computational details**: The Orca program was used for all calculations.^[25]^ Geometry optimizations of the 126 atom models were performed in their respective high‐spin states using the B3LYP^[26]^ functional and the conductor‐like polarizable continuum model (CPCM)^[27]^ with a dielectric constant of 6 to approximate the polarizability of the protein environment. Through this study the zero‐order regular approximation (ZORA)^[28]^ was used to include scalar relativistic effects. Also, the ZORA‐TZVP all‐electron basis sets^[29]^ were used throughout for all atoms except C and H, for which the ZORA‐SVP basis sets were used. Tight convergence criteria (TightSCF in Orca convention) and increased angular integration grids (“Grid5” and “GridX7” in ORCA convention) and radial integration grids (“IntAcc 6.0”) were used in all calculations. In order to speed up the calculations the resolution of identity (RI) approximation for the Coulomb integrals and the chain of spheres approximation for exchange were employed.^[30]^ For the single‐point energy calculations the nonstandard B3LYP and TPSSh^[31]^ functionals were used with varying degrees of Hartree−Fock exchange. Single‐point DFT calculations for the 126 atom models were carried out without CPCM solvation, so that the results are directly comparable with DLPNO‐CCSD(T) derived values.

For the geometry optimization of the large 325 atom models the TPSSh functional was used along with CPCM solvation with a dielectric constant of 6. The optimization was performed at the *αααβ* (Mn1 Mn2 Mn3 Mn4) spin configuration for **S_3_O** and at the *βααα* spin configuration for **S_3_P**. The high‐spin and broken‐symmetry optimized geometries are essentially indistinguishable, in line with past experience on high‐valent manganese systems. Specifically the energetic effect of geometric relaxation on the relative energies of the two isomers was determined to be at most 0.5 kcal mol^−1^, and hence it does not affect the analysis and conclusions of this work. Entropic contributions are not included. The effect of dispersion corrections was considered using Grimme's latest D4 atomic‐charge dependent dispersion corrections.^[32]^

For the calculation of exchange coupling constants *J_ij_*, the single point energies of the high‐spin and all possible broken‐symmetry solutions were calculated using the broken‐symmetry DFT (BS‐DFT) methodology with the TPSSh functional (Table S4). Convergence to the correct spin state was confirmed by the Mn spin populations. Subsequently, the six unknown *J_ij_* constants were determined using singular value decomposition. Finally, the computed *J_ij_* constants were used for the diagonalization of the Heisenberg‐Dirac‐Van Vleck Hamiltonian [Eq. (1)] in order to derive the complete set of spin eigenstates.

(1) $$\hat{H}=\;-2\;\sum_{i<j}J_{ij}\;{\hat{S}}_{i\;}{\hat{S}}_{j\;}$$

This methodology has been detailed in several studies and used successfully for a large number of oligonuclear exchange‐coupled Mn systems.^[33]^

The wave function‐based calculations were carried out using the domain‐based local pair natural orbital coupled cluster method with singles, doubles, and perturbative triples excitations, DLPNO‐CCSD(T). Different definitions of the perturbative triples term will be discussed in the following. Unrestricted Kohn–Sham B3LYP orbitals were used as input for the DLPNO‐CCSD(T) calculations. The DLPNO‐CCSD(T) calculations were performed in the single‐reference high‐spin states (*S*=6 for **S_3_O** and *S*=7 for **S_3_P**). Two basis set combinations were used for the two‐point extrapolations (see Supporting Information for details); TZ/TZ: ZORA‐def2‐TZVP on all atoms, except for C and H where the ZORA‐def2‐SVP basis sets were used, and QZ/TZ: ZORA‐def2‐QZVPP on Mn ions, ZORA‐def2‐TZVP on N, O, Ca and Cl, and ZORA‐def2‐SVP for C and H.[^29^, ^34^] Corresponding auxiliary /C and /J basis sets were used where applicable.[^34^, ^35^]

The correlation energy components were extrapolated to the complete PNO space limit by using Equation 2:

(2) $$E^{x}=\;E^{\infty }+A\cdot x^{-\beta }$$

where $E^{x}$ is the correlation energy calculated with *T*_CutPNO_=10^−*x*^, $E^{\infty }$ is the extrapolated correlation energy at the PNO space limit and A and β are constants. This expression leads to an extrapolation equation analogous to the two‐point extrapolation scheme for the complete basis set (CBS) limit [Eq. 3]:^[36]^

(3) $$E^{\infty }=\frac{y^{\beta }{\cdot E}^{y}-\;x^{\beta }{\cdot E}^{x}\;}{y^{\beta }-\;x^{\beta }}$$

Using a constant *F* defined as [Eq. 4]:

(4) $$F=\;\frac{y^{\beta }}{y^{\beta }-\;x^{\beta }}$$

Equation 2 can be expressed as [Eq. 5]:

(5) $$E^{\infty }=E^{x}+F\;\cdot \;\left(E^{y}-E^{x}\right)$$

The recommended^[36]^ value for *F* is 1.5, as it has been determined to give optimal results for large benchmark sets. Using three different *T*_CutPNO_ thresholds at both NormalPNO and TightPNO settings, we plotted fits of the correlation energy as a function of x , based on Equation (2) (Figure S3). The determined *F* values are within the 1.51–1.66 range. Given that these values are close to the more widely benchmarked recommended value of 1.5, for the sake of standardization we used *F*=1.5 for the PNO space extrapolations.

## Results and Discussion

### Structural features and magnetic properties

Starting from the latest crystallographic model of the S_3_ state of the OEC, computational models were constructed containing the complete first coordination sphere amino acids and water molecules (W1–W4) as well as selected second coordination sphere water molecules and the hydrogen‐bonding D1‐His337 in its protonated form.^[37]^ W2 was assigned as hydroxo in the oxo‐hydroxo isomer^[33f]^ and as aquo in the peroxo isomer,^[14]^ which ensures that the two models are exact isomers and hence directly comparable in terms of energetics. The form of W2 in the peroxo structure has been addressed by Corry and O'Malley, who presented^[20]^ a deprotonated model of S_3_‐state peroxo isomer and calculated an *S*=4 ground state, which does not agree with the *S*=3 species observed experimentally. The models used for the coupled‐cluster calculations are sufficiently large to be representative of the electronic structure of the OEC and to naturally adopt realistic protein‐like geometries upon optimization (indeed they are comparable in size to what was considered standard for DFT models of the OEC and adequate for spectroscopic investigations a few years ago[^33a^, ^38^]), while pushing the limits of feasibility of DLPNO‐CCSD(T) calculations on current cutting‐edge computing facilities available to us.

The structures were optimized either as oxo‐hydroxo (**S_3_O**) or as peroxo (**S_3_P**) forms in their respective high‐spin configuration using the B3LYP functional. The most important calculated interatomic distances of the derived structures are shown in Table S1, where comparisons of key structural parameters with PDB IDs: 5WS6 (Suga et al. 2017),^[4b]^ 6JLL (Suga et al. 2019),^[4c]^ and 6DHO (Kern et al. 2018)^[4d]^ XFEL models as well as with EXAFS derived Mn−Mn distances^[39]^ are also presented. The calculated spin populations (Table S2) confirm the IV–IV‐IV–IV and III–IV‐IV–III valence distribution for the Mn1−Mn4 ions in the oxo‐hydroxo and peroxo isomers, respectively. The Jahn‐Teller axes of the Mn1^III^ and Mn4^III^ ions in the peroxo structure are collinear, as in one of the Jahn‐Teller isomeric forms of the resting S_1_ state.^[40]^ According to the root mean square deviations (RMSDs) from the XFEL distances (Table S3), the structure that corresponds to the **S_3_P** isomer is in best agreement with the 5WS6 XFEL model, which has been directly interpreted as a peroxo form,^[4b]^ with an RMSD of 0.21 Å for the 25 atom core structure and 0.12 Å for the Mn atoms only, while the **S_3_O** models show similar level of RMSD with respect to all XFEL models associated with the S_3_ state. In particular, the O5−O6 distance of 1.4 Å observed for **S_3_P** is identical with the 5WS6 crystallographic model, whereas the O5−O6 distance of 2.5 Å calculated for **S_3_O** is much larger than that observed in the XFEL structures. By contrast, optimized Mn−Mn distances of the **S_3_O** isomer are more consistent with EXAFS spectroscopy than **S_3_P**, due to the larger Mn3‐Mn4 distance in **S_3_P**, consistently with previous observations.^[2c]^ Based on the calculated exchange coupling constants *J_ij_* (Table S5) the dominant ground state spin configurations of **S_3_O** and **S_3_P** are *αααβ* and *βααα*, respectively, and both forms adopt *S*=3 ground states. Notably, geometric relaxation of **S_3_O** and **S_3_P** in their lowest‐energy broken‐symmetry states has negligible effect on the structural parameters and relative energies of the models. From the above results, we conclude that the small OEC models are adequate to describe the basic features of the S_3_ isomers under investigation.

### Redox isomerism energetics from DLPNO‐CCSD(T)

Having established the respective structural models, we proceed with the primary objective of the present work, which is to perform DLPNO‐CCSD(T) calculations to extract reliable and reasonably converged relative energies for the two forms. The electronic energy difference, Δ*E*_PO_, between the S_3_‐hydroxo (**S_3_O**) and S_3_‐peroxo (**S_3_P**) isomers, used throughout this work is defined as [Eq. 6]:

(6) $$\Delta E{}_{\mathrm{PO}}=E\left(S{}_{3}P\right)-E\left(S{}_{3}O\right)$$

The results of the DLPNO‐CCSD(T) calculations are given in Table 1. The correlation energy contributions of single and double excitations and perturbative triples (T_0_) and (T_1_) corrections as well as the contribution of each correlation method to the relative energy, Δ*E*_PO_ are presented. Single‐point DLPNO‐CCSD(T) calculations were performed with different levels of approximation (i. e., with various *T*_CutPairs_, *T*_CutPNO_ and *T*_CutDO_ thresholds using LoosePNO, NormalPNO and TightPNO settings in Orca), in order to explore the convergence behavior of the method on the absolute energies of the OEC models and on their relative energy, Δ*E*_PO_ (see Table S6 for a detailed presentation of the results).

**Table 1:** Contributions of the Hartree−Fock (HF) and correlation energy to the energy difference, Δ*E*_PO_, between the S_3_‐hydroxo (**S_3_O**) and S_3_‐peroxo (**S_3_P**) forms. All values in kcal mol^−1^.

				Correlation energy contributions to Δ*E*_PO_				
	Basis set	*T*_CutPNO_	HF	CCSD	LMP2	(T_0_)	(T_1_)	Δ*E*_PO_^[a]^
NormalPNO	TZ/TZ	1.0×10^−6^	−103.82	94.21	2.08	35.07	40.77	33.25
	TZ/TZ	1.0×10^−7^	−103.82	93.79	2.38	35.89	41.79	34.14
	TZ/TZ	3.33×10^−7^	−103.82	94.22	2.61	35.39	41.19	34.21
	QZ/TZ	3.33×10^−7^	−101.40	93.66	4.35	36.58	42.26	35.95
		$\delta^{CBS}$	2.76	−0.57	1.74	2.07	1.86	5.79
	CBS/TZ	Inf. PNO	−101.06	93.01	2.38^[b]^	38.36	44.16	38.49
TightPNO	TZ/TZ	1.0×10^−6^	−103.82	93.24	0.43	35.44		
	TZ/TZ	1.0×10^−7^	−103.82	93.89	0.82	36.33		
	CBS/TZ	Inf. PNO	−101.06	93.64	0.82^[b]^	38.84		37.56^[c]^

[a] Estimated as the sum of SD, MP2 and (T_1_) values. [b] LMP2 was not extrapolated to the CBS limit. [c] Estimated using the (T_1_) contribution from the NormalPNO calculation according to Equation (7).

We investigated the dependence of the DLPNO‐CCSD(T) correlation energy on the dimension of the PNO space, described by the *T*_CutPNO_ threshold, which is the most important threshold for the accuracy of the method. Additionally, we approached the basis set limit of the DLPNO‐CCSD(T) calculations with respect to the metal ions. Selective increase of the basis set size used to describe the metal ions significantly enhances the accuracy of the method,^[41]^ while the effect of increasing the size of the ligands basis set has minimal effect. We employ a two‐point extrapolation scheme using two basis set combinations; ZORA‐def2‐TZVP on all atoms, except C and H where ZORA‐def2‐SVP was used (denoted TZ/TZ on Table 1) and ZORA‐def2‐QZVPP on Mn ions, ZORA‐def2‐TZVP on O, N, Ca, Cl and ZORA‐def2‐SVP on C and H (denoted QZ/TZ on Table 1). The validity of extrapolation using def2 basis sets has been confirmed previously.^[42]^ The extrapolated energy with respect to i) Mn CBS limit and ii) PNO space limit for the DLPNO‐CCSD(T) correlation energy is estimated according to the formula [Eq. 7]:

(7) $$E=\;E_{HF}^{CBS}+E_{SD}^{\infty }+\;\delta_{SD}^{CBS}+\;E_{\left(T_{1}\right)}^{\infty }+\;\delta_{\left(T_{1}\right)}^{CBS}$$

where $E_{HF}^{CBS}$ the HF energy extrapolated to the CBS limit according to Equation (S1), $E_{SD}^{\infty }$ and $E_{\left(T_{1}\right)}^{\infty }$ are the DLPNO‐CCSD and (T_1_) contributions to the correlation energy, respectively, extrapolated to the infinite PNO space limit according to Equation (5) and $\delta_{SD}^{CBS}$ and $\delta_{\left(T_{1}\right)}^{CBS}$ are additive corrections for the incompleteness of the basis sets used, defined in equation S3.

In the extrapolated results using NormalPNO settings, inclusion of only singles and doubles excitations (DLPNO‐CCSD) is only able to recover 95.0 and 95.3 % of the total correlation energy including the perturbative triples correction, for the **S_3_O** and the **S_3_P** structures, respectively. It is apparent that extensive retrieval of correlation energy is required in order to provide a reliable energy profile. Inclusion of the semicanonical triples, denoted (T_0_), recovers an additional 4.4 % of the correlation energy for **S_3_P**, but 4.6 % for **S_3_O**. The more accurate perturbative triples correction where the triples amplitudes are computed iteratively, denoted (T_1_), show additional stabilization for the **S_3_O** form, leading to a final Δ*E*_PO_ value of 38.5 kcal mol^−1^.

Upon inspection of the results (Tables 1 and S6), as the *T*_CutPNO_, *T*_CutPairs_ and *T*_CutDO_ thresholds get tighter, the SD, (T_0_) and (T_1_) retrieved correlation energies increase. The increase is larger for the low spin **S_3_O** structure, which results in a larger Δ*E*_PO_ value as the accuracy of the method is enhanced. The (T_0_) and (T_1_) contributions to the Δ*E*_PO_ value consistently increase, starting from 32.5 and 36.8 kcal mol^−1^, respectively, with the LoosePNO settings (Table S6) and reaching 35.9 and 41.8 kcal mol^−1^, respectively, with the NormalPNO settings with *T*_CutPNO_ 1×10^−7^ (Table 1). Conversely, the SD correlation energy shows negligible change in Δ*E*_PO_ as the accuracy of the method is improved. Only the electron pairs which are characterized as strongly correlated by local second‐order many‐body perturbation theory (LMP2), “strong pairs” in Orca convention, are treated at the coupled cluster level. For the “weak pairs”, which are expected to contribute negligibly to the total correlation energy, the corresponding LMP2 energy is added to the CCSD correlation energy to give the final DLPNO‐CCSD energy (SD). As the *T*_CutPNO_ threshold decreases, more electron pairs are treated as “strong pairs”, leading to increase in the CCSD correlation energy and decrease in the LMP2 energy attributed to reduced number of “weak pairs”. We observed that the SD=CCSD+LMP2 correlation energy is slightly decreased when *T*_CutPNO_ threshold increases. This is possibly due to a slight overestimation of the LMP2 correlation energy, which however, compensates for the perturbative triples correlation energy. Even though the error is very small ∼0.001 %, it leads to non‐negligible error in the calculated Δ*E*_PO_ values, by overstabilizing the **S_3_O** form. Based on the above observations, the most reliable Δ*E*_PO_ estimate is 37.6 kcal/mol, derived from the CCSD correlation energy contribution from the PNO space extrapolated DLPNO‐CCSD/TightPNO/Mn CBS limit calculation and the perturbative triples (T_1_) correlation energy contribution from the PNO space extrapolated DLPNO‐CCSD/NormalPNO/Mn CBS limit calculation, according to the equation [Eq. 8]:

(8) $${\Delta E}_{PO}=\;{\Delta E}_{HF}^{CBS}+{\Delta E}_{CCSD/TightPNO}^{\infty PNO}+\;\delta_{CCSD}^{CBS}+{\Delta E}_{LMP2/TightPNO}^{x=7}+{\Delta E}_{\left(T_{1}\right)/NormalPNO}^{\infty PNO}+\;\delta_{\left(T_{1}\right)}^{CBS}$$

The LMP2 correlation energy was not extrapolated with respect to the PNO space, because in the PNO space limit zero pairs will be characterized as “weak”. The TightPNO value of the calculation with the tighter *T*_CutPNO_ threshold *x*=7 (that involves the lowest number of “weak” pairs) is used instead. In addition, the LMP2 correlation energy was not extrapolated to the CBS limit.

**Figure 3:**
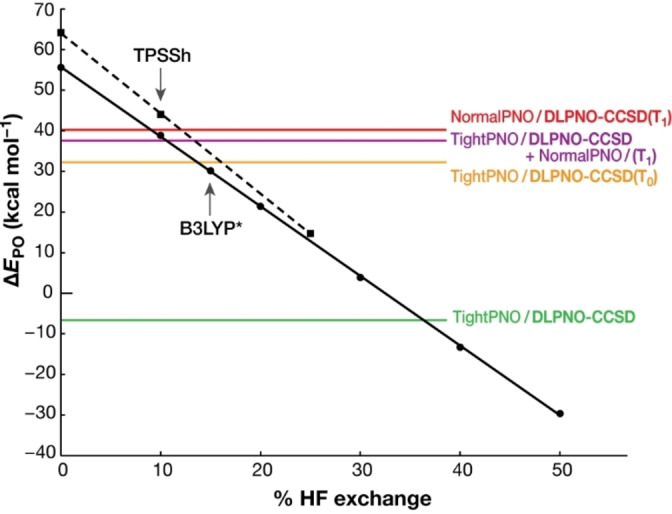
Energy difference between **S_3_P** and **S_3_O** structures, Δ*E*_PO_, by the B3LYP functional with different HF exchange percentages from 0 to 50 % (circles and solid black line) and by the TPSSh functional with 0, 10 and 25 % HF exchange (squares and dashed line). The DLPNO‐CC‐calculated Δ*E*_PO_ values are shown in the colored horizontal lines; in green the DLPNO‐CCSD relative energy calculated with TightPNO settings in the CBS and infinite PNO space limit, in orange the contribution of the perturbative triples correction (T_0_) has been added to the previous DLPNO‐CCSD relative energy, in red the DLPNO‐CCSD(T_1_) relative energy calculated with NormalPNO in the CBS and infinite PNO space limit, and in purple the most reliable Δ*E*_PO_ estimate of this work, calculated as the sum of the TightPNO DLPNO‐CCSD and the iterative triples (T_1_) contribution using NormalPNO settings in the CBS and infinite PNO space limit.

The DLPNO‐CC derived values for Δ*E*_PO_ extrapolated to the CBS limit and to the PNO space limit are shown in Figure 3. Notably, the extrapolated Δ*E*_PO_ values calculated at the DLPNO‐CCSD(T_0_)/TightPNO and DLPNO‐CCSD(T_0_)/NormalPNO levels of theory are almost identical (32.2 and 32.7 kcal mol^−1^, respectively). This is because the slight overestimation of the LMP2 correlation energy in the NormalPNO settings makes up for the correlation energy that is retrieved with tighter PNO cutoffs.

A technical aspect related to the presently employed theoretical approach is the multireference nature of the wave function that describes the ground state spin configuration of the OEC models. Based on the calculated pairwise exchange coupling constants for **S_3_O** and **S_3_P** models, the ground state spin configurations are *αααβ* and *βααα*, respectively, and the ground state spin estimated by diagonalization of the spin Hamiltonian is *S*=3 for each model (Table S5). Therefore, the magnetic ground states of both systems are intrinsically multireference in character. This problem is circumvented by performing the DLPNO‐CCSD(T) calculations using the high‐spin determinant of each S_3_ isomer, that is, *S*=6 for **S_3_O** and *S*=7 for **S_3_P**. As expected for high‐valent manganese systems, both the T_1_ diagnostic and the largest excitation amplitudes show that none of the isomers has multireference character in the high‐spin states. The *S*=6 excited state for **S_3_O** is 241 cm^−1^ higher than the ground state and the *S*=7 state for **S_3_P** is 428 cm^−1^ higher, thus the energy difference between the ground state and the state of highest spin is only about 0.5 kcal mol^−1^ higher for **S_3_P** than for **S_3_O**. This means that the DLPNO‐CCSD(T) Δ*E*_PO_ value computed for the respective high‐spin states is directly transferable to the two redox isomers in their *S*=3 ground states.

At this point we should consider possible limitations of the methodology presented in this study. A possible source of error may relate to the extrapolation to the CBS limit are the size of the basis sets used, as well as the fact that CBS extrapolation was performed only with respect to the Mn ions. Additional errors may arise from the DLPNO approximation itself[^20e^, ^43^] or from the inadequate convergence of the coupled cluster expansion at the CCSD(T) level.[^20e^, ^44^] In this work, the use of the (T_1_) approach and the extrapolation to the complete PNO space mitigate some, but not all sources of error that were present in past studies. Nevertheless, even in the presence of such possible uncertainties, the DLPNO‐CC approach provides the maximum attainable accuracy for the system under consideration, while the magnitude of the energy difference offers strong confidence that the conclusions are qualitatively definitive. The net result is that **S_3_O** is more stable than **S_3_P** by 37.6 kcal mol^−1^ for the present models.

### Evaluation of density functional theory

To study the effect of Hartree‐Fock exchange in the calculated energy difference, a set of single point DFT calculations using functionals with varying contribution of exact exchange were performed. Δ*E*_PO_ calculated using B3LYP (20 % HF exchange) in the respective high spin configurations (*S*=6 for **S_3_O** and *S*=7 for **S_3_P**) is 21.4 kcal mol^−1^. Single point energy calculations of Δ*E*_PO_ using the B3LYP and TPSSh functionals with different HF exchange percentages give a wide range of Δ*E*_PO_ values, which are plotted in Figure 3. From this diagram we observe that the stability of the peroxo form consistently increases when the HF percentage increases. At the limit of 100 % HF exchange Δ*E*_PO_ is −101 kcal mol^−1^, indicating a gross overstabilization of the peroxo form. This is consistent with the observation that *E*(HS)−*E*(LS) energy difference values generally increase with the degree of treatment of electronic correlation, because electron correlation is more important in lower‐spin states.^[15f]^ Thus, the relative stability of the oxo‐hydroxo and peroxo forms cannot be even qualitatively predicted, since the calculation is very sensitive to the HF exchange percentage. This reflects similar observations reported previously for the OEC[^13b^, ^20^] as well as for other systems.[^15a^, ^15b^, ^15c^] In general, calculations of relative energies between species with different numbers of unpaired electrons using DFT do not benefit from the more or less systematic error cancellation that is encountered for isomers with the same electronic configuration. It is also the case here that errors in DFT do not systematically cancel out when the energy difference between the peroxo form (14 unpaired electrons) and the oxo‐hydroxo form (12 unpaired electrons) is calculated.^[24a]^

It appears that the non‐standard B3LYP functional with approximately 10 % HF exchange percentage gives the closest Δ*E*_PO_ estimate to the DLPNO‐CCSD(T_1_) calculation (Figure 3, purple horizontal line). The 15 % fraction (B3LYP*) gives an error of 8 kcal mol^−1^ with respect to the DLPNO‐CCSD(T) value, in favor of the **S_3_P** (Figure 3). By contrast, the TPSSh functional^[31]^ gives an error of 6 kcal mol^−1^ in favor of **S_3_O**. Even though the B3LYP* functional has shown superior performance in many cases,[^15a^, ^15g^, ^45^] the admixture of exact exchange is not the only determining factor, as the effects of the underlying functional^[16b]^ and dispersion corrections^[46]^ can be significant, and sometimes a lower percentage of exact exchange or distinct functional forms are preferred.^[47]^ This underlines the non‐universality problem of DFT for spin state energetics and the need for calibration using methods of higher accuracy. The TPSSh functional (10 % HF exchange) has been reported to perform best at the calculation of magnetic properties^[48]^ and at the calculation of the energy differences between high spin and low spin states of Mn^III^ spin crossover complexes,^[49]^ where other functionals showed poorer performance on the same tasks. Additionally, in previous studies of Photosystem II, it has been reported that the B3LYP functional with 15 % HF exchange, B3LYP*,^[15a]^ reproduces experimental redox energetics well.[^2g^, ^11c^] Given that TPSSh and B3LYP* bracket what is arguably the best possible estimate of Δ*E*_PO_ achievable by modern quantum chemistry for this system, we expect that the results using these functionals will be equally reliable for larger models of these isomeric forms where the composition and electronic structure of the inorganic core remain identical to the medium‐sized models discussed above.

### Effect of longer‐range protein matrix interactions

After achieving convergence with respect to the accuracy of the wavefunction method used to calculate the respective correlation energies of the compact OEC models, we examined how the inclusion of additional, more remote second‐sphere elements of the protein matrix into the QM model might affect the energetics. We constructed 325 atom S_3_‐state models starting from the 6JLL (monomer A) crystal structure. The structures were optimized in the lowest broken‐symmetry spin configurations using the TPSSh functional. TPSSh gives an energy difference Δ*E*_PO_ of 18.7 kcal mol^−1^ and single point calculations using B3LYP with 10 % HF exchange gives Δ*E*_PO_ of 17.6 kcal mol^−1^. Dispersion corrections with the D4 model at the TPSSh level used for geometry optimizations contribute to a small differential stabilization of **S_3_P** by 1.1 kcal mol^−1^, thus we arrive at a combined best estimate of 16.4 kcal mol^−1^ for the energy difference Δ*E*_PO_. This result is close to the value of ∼15 kcal mol^−1^ reported by Isobe et al. using B3LYP with 10 % HF exchange for models of similar size.^[13b]^ We observe that inclusion of peripheral amino acids in the larger models leads to greater stabilization of **S_3_P** with respect to **S_3_O**, which can be attributed to the Jahn‐Teller distorted Mn1^III^ and Mn4^III^ ions of **S_3_P** that need a larger and more flexible protein backbone to optimize Mn−O bond distances. Additional long‐range electrostatic protein matrix effects could be considered within a QM/MM framework, but past convergence studies suggested that their effect on the local electronic structure of the inorganic cluster is limited.^[50]^

In conclusion, second‐shell effects selectively stabilize the **S_3_P** form, but this remains considerably higher in energy than the oxo‐hydroxo form **S_3_O**.

### Implications for experimental interpretations and for the mechanism of water oxidation

The proposed “early onset” O−O bond formation mechanism between O5 and O6 in the S_3_ state requires the existence of an equilibrium between the oxo‐hydroxo and peroxo forms, achieved by intramolecular proton transfer from O6 to W2 concerted with two one‐electron transfer steps from O5 and O6 to Mn4 and Mn1, which leads to Mn^IV^→Mn^III^ reductions.[^14^, ^20^] For this process to be feasible, the two forms must be close in energy. X‐ray absorption spectroscopy (XAS)^[51]^ and X‐ray emission spectroscopy (XES) experiments^[9]^ as well as EPR studies[^3a^, ^5a^, ^5d^] are in favor of a IV–IV–IV–IV valence distribution. In addition, Corry and O'Malley reported^[20]^ that the calculated ^55^Mn hyperfine coupling constants for a peroxo model do not agree with experiment, in contrast to the oxo‐peroxo form. Thus, assuming both forms were accessible, one would expect that the oxo‐hydroxo form must be lower in energy than the peroxo form, even though this cannot constrain the Δ*E*_PO_ value, as EPR experiments were carried out at low temperatures. The calculated energy difference of 16.4 kcal mol^−1^ indicates that either the peroxo structure is not formed in the ground state of S_3_ or it exists in negligible quantities. The second hypothesis would be consistent with the suggestion^[2n]^ that a high‐energy peroxide isoform of S_3_ can be preferentially oxidized by the transiently formed tyrosyl radical after the final light‐driven oxidation step of the cycle (S_3_Y_Z_^.^ state), so that the O−O bond is formed prior to formal S_4_‐state formation by a radical coupling mechanism, which potentially lowers the activation barrier. In either case, the peroxo isomer *cannot* be observable during experimental characterization of S_3_ in the ground state. Here lies the paradox with the current XFEL models; the disagreement between these models and the spectroscopic as well as computational results leads us to the conclusion that the available XFEL crystallographic models of the S_3_ state are far from definitive. Uncertainties in these structural studies with respect to the OEC structure in the S_3_ state are associated not simply with the low overall resolution, but also with the specific uncertainties in the positions of the light O atoms, the quantification of S_*i*_ state conversion, ambiguities in analysis and interpretation of experimental data, the effect of dark adaptation of the samples, and the persisting possibility of partial reduction.[^2c^, ^8a^, ^52^]

Formation of metal peroxo intermediates is a common feature of metalloenzymes that catalyze O_2_‐dependent reactions,^[17c]^ often involving intermediates with a M_2_O_2_^*n*+^ core in their active sites (M=Mn, Fe or Cu).^[53]^ O_2_ activation is achieved through concerted electron flow between the M−O bonds of the cluster along with structural changes, therefore to understand the chemical reactivity of high‐valent metal‐oxo bonds, one needs to accurately describe their electronic structure.[^2d^, ^10a^, ^54^] The electronic rearrangement accompanying the **O**⇌**P** redox isomerism, that is, the equilibrium between peroxo with reduced metal ions and bis(oxo) with high‐valent metal ions, is a typically challenging process for DFT due to involvement of different spin states and configurations.^[55]^ Wave function based methods are crucial for evaluating whether the metal has enough oxidative ability to draw electron density from an oxo moiety or if it can “inject” electrons to the O_2_ antibonding orbitals to break the O−O bond. The Mn1‐O6‐O5‐Mn4 group in the S_3_‐state of the OEC resembles two mononuclear Mn complexes forming a possible **O**⇌**P** equilibrium, similar to other metalloenzymes. The results of the present work establish that this equilibrium, if at all relevant for the S_3_ state of the OEC, is very strongly shifted to the left.

This makes sense in the context of biological water oxidation because it enables the enzyme to avoid formation of undesirable, and possibly damaging, partially oxidized products. Therefore, the Mn^IV^_4_ oxo‐hydroxo form **O** should be a better representation of the S_3_ state and possibly the predominant species, which confirms the spectroscopic conclusion that the S_2_→S_3_ transition must involve Mn‐centered rather than ligand‐centered oxidation. This contradicts the literal interpretations of XFEL models that have been evolving in the last few years and evidently have plenty of room for further improvement and refinement. This conclusion stands independently of the likely equilibrium between various all‐Mn^IV^ forms comprising the heterogeneous S_3_ state, which might be affected among others by the extent of hydration^[3a]^ and by the protonation state of the tyrosyl radical.[^2k^, ^56^] An important question that remains entirely open is whether metal‐based storage of three oxidizing equivalents is sufficient to trigger O−O bond formation in the active site of the OEC, or one more light‐induced metal based oxidation is needed to proceed to O−O bond formation in the transient S_4_ state. More experimental information both on the S_3_ state and on the nature of the S_3_Y_Z_^.^ metalloradical intermediate will be required to address this point.

## Conclusions

In this work, we applied the DLPNO‐CCSD(T) method to derive accurate reference values for the relative energy of oxo‐hydroxo and peroxo forms of the S_3_ state of the oxygen‐evolving complex. Within the DLPNO‐CCSD(T) framework, we investigated the convergence of the different correlation energies, that is, single and double excitations, SD, semicanonical perturbative triples, (T_0_), and iterative perturbative triples, (T_1_), with respect to the basis set and to the *T*_CutPNO_ threshold, which is considered to be the most important for the accuracy of the method. Despite the “black box” character of the method in terms of performing the calculations, one should expend dedicated effort to carefully examine the convergence behavior of the method and evaluate the effect of the various cutoffs in order to reach the maximum attainable accuracy and efficiency. Nevertheless, this remains a promising approach for similar systems and problems, while additional algorithmic improvements or multilayer implementations can serve to expand the applicability of the method. We therefore expect further applications to the energetics of large biologically relevant molecules where species with different numbers of unpaired electrons are involved. Our evaluation of DFT against the coupled cluster results for the case of the S_3_ state isomers supports the use of hybrid functionals with low exact exchange for such systems. In terms of the chemical nature of the system under study, this work reaches the clear conclusion that the oxo‐hydroxo model is strongly stabilized energetically compared to the peroxo formulation for the final metastable S_3_ state of the OEC. This disfavors the possibility of Mn reduction in the S_2_→S_3_ transition and, by extension, the formation of an O−O bond to any considerable extent in the S_3_ state. Other proposed S_3_ redox isomers include ligand‐based radical forms such as the superoxo and the oxyl‐oxo formulation, which are not investigated in this work. Although the former is not a credible candidate for the S_3_ state, the latter is a possibility that should be considered more closely.[^4c^, ^13b^] The applicability of DLPNO‐CCSD(T) to such metalloradical intermediates is questionable, owing to their multireference character or the necessity to approximate their electronic structure via broken‐symmetry determinants.^[13b]^ Therefore, alternative high‐level theoretical methods should be considered in the future.[^20c^, ^57^] These new results do provide strong evidence against early‐onset O−O bond formation in the OEC, and therefore the highest‐level available quantum chemical results on energetics are fully aligned with all available spectroscopic observations on the S_3_ state. This serves as a warning against literal interpretations of the various geometric models inferred from XFEL crystallographic studies, particularly after the compelling demonstrations of crystallographically unresolved heterogeneity in the S_3_ state by EPR spectroscopy.

## Conflict of interest

The authors declare no conflict of interest.

## Supporting information

As a service to our authors and readers, this journal provides supporting information supplied by the authors. Such materials are peer reviewed and may be re‐organized for online delivery, but are not copy‐edited or typeset. Technical support issues arising from supporting information (other than missing files) should be addressed to the authors.

Supporting Information
