# Supplementary material for: Redox Isomerism in the S3 State of the Oxygen‐Evolving Complex Resolved by Coupled Cluster Theory
Source: Chemistry. 2021 Aug 6;27(50):12815–25. doi: 10.1002/chem.202101567 (PMC8518824; doi:10.1002/chem.202101567)
Supplement: Supplementary file 1 — Supporting Information [file CHEM-27-12815-s001.pdf]

Chemistry–A European Journal

Supporting Information

Redox Isomerism in the S_3 State of the Oxygen-Evolving Complex Resolved by Coupled Cluster Theory

Maria Drosou and Dimitrios A. Pantazis*

Contents

Structural Parameters of Optimized Models 2

Magnetic Properties 5

DLPNO-CCSD(T) Methodology 6

Extrapolation to the Complete Basis Set Limit 7

Assessment of Multireference Character 7

Detailed DLPNO-CCSD(T) Results 8

References 12

Cartesian Coordinates of Optimized Structures 13

Structural Parameters of Optimized Models

Table S1. Selected interatomic distances (Å) of the optimized 126-atom and 325-atom S_3-state models presented in this work compared with XFEL and with EXAFS derived structural parameters.

	Model	Mn1-Mn2	Mn1-Mn3	Mn1-Mn4	Mn2-Mn3	Mn3-Mn4	Mn1-Ca	Mn2-Ca	Mn3-Ca	Mn4-Ca	Mn1-O6	Mn4-O5	O5-O6	Ref.
126 atom models	**S_3O**	2.79	3.63	5.40	2.85	2.76	3.46	3.45	3.62	4.11	1.81	1.81	2.52	This work
	**S_3P**	2.81	3.41	5.30	2.78	2.95	3.47	3.45	3.58	4.14	2.15	2.27	1.43	
325 atom models	**S_3O**	2.77	3.62	5.32	2.83	2.80	3.44	3.38	3.51	4.02	1.78	1.82	2.45	This work
	**S_3P**	2.85	3.45	5.23	2.72	2.97	3.47	3.40	3.53	4.04	1.94	2.30	1.45	
	**5WS6**[a]	2.63	3.25	5.03	2.79	2.74	3.43	3.39	3.53	4.04	2.26	2.30	1.45	[1]
	**6DHO**[a]	2.75	3.33	5.06	2.84	2.77	3.37	3.33	3.56	4.00	1.79	2.22	2.09	[2]
	**6JLL**[a]	2.58	3.31	5.21	2.71	2.90	3.50	3.46	3.50	3.98	1.71	2.20	1.90	[3]
	**EXAFS**	[2.75, 2.75, 2.79, 3.26]					[3.37, 3.99]							[4]
	**EXAFS**	[2.76, 2.78, 2.78, 3.50]												[5-6]

[a] Average distances between A and a monomers.

Geometry optimizations of the 325-atom models were also performed in their respective deprotonated forms, i.e. with W2=OH for **S_3P** and W1=OH for **S_3O**. The calculated electronic energy values for the deprotonation of **S_3O** and of **S_3P** are 302.5 and 300.1 kcal mol^{-1}, respectively, therefore the calculated ΔE_{PO} value for the deprotonated forms is less than 2.5 kcal mol^{-1} smaller compared to the protonated structures. The results suggest that the acidity of the OEC environment would affect

both isomers equally, therefore the conclusions of this work are unaffected by assumptions regarding the protonation state of terminal water ligands.

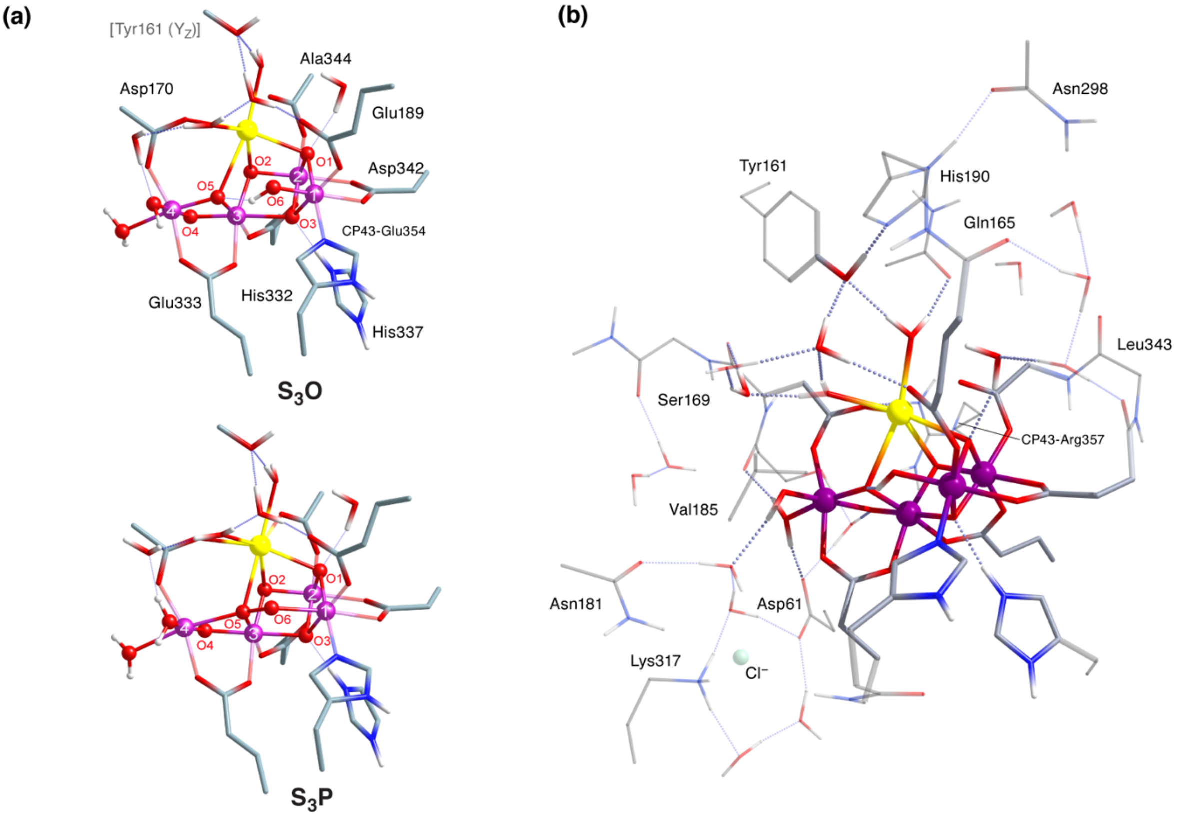

Figure S1. (a) Structures of the 126-atom **S_3O** and **S_3P** models. (b) Structure of the 325-atom model (the oxo-hydroxo isomer is depicted). The thick lines correspond to the substructure used in the 126-atom models.

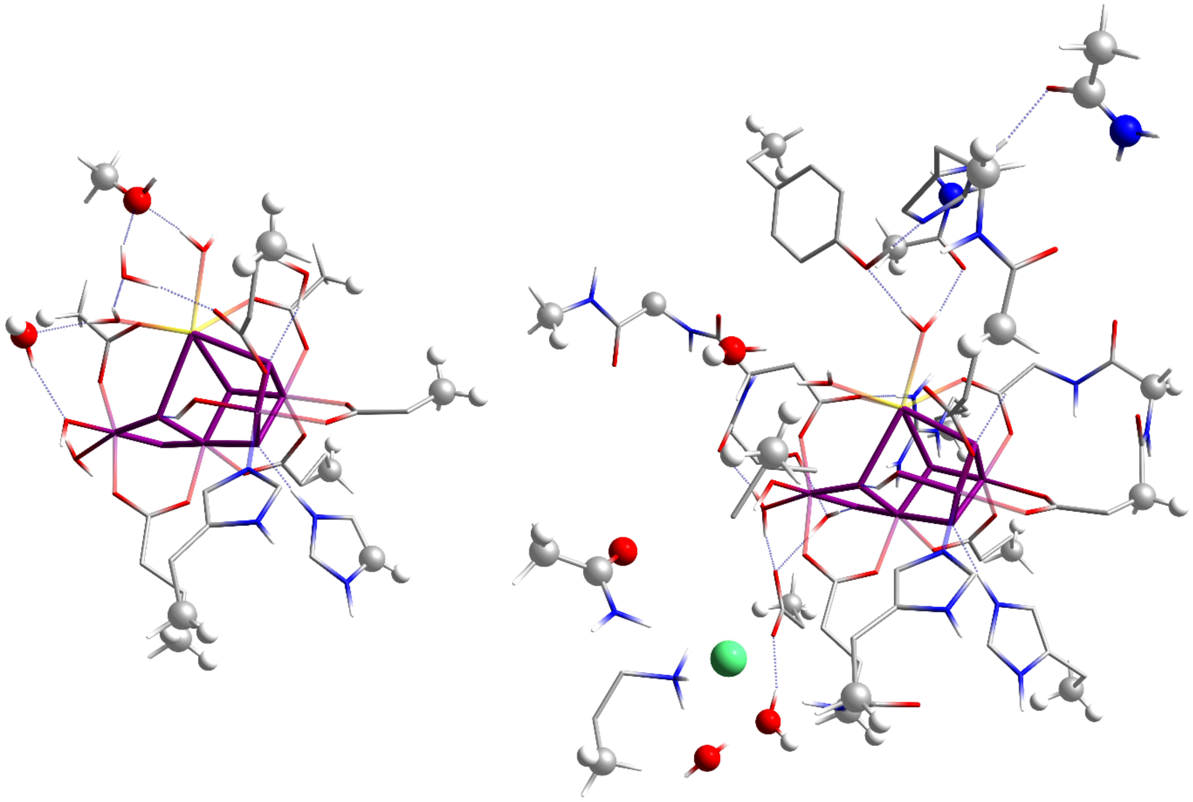

Figure S2. The 126- (left) and 325- (right) atom models used in this study, with the constrained atoms shown as spheres. Most hydrogen atoms and non-coordinating waters (in the larger model) have been omitted for clarity.

Table S2. Computed spin populations of the 126-atom and 325-atom models.

		Spin populations (TPSSh)			
	Model	Mn1	Mn2	Mn3	Mn4
126 atom models	S_3O	2.87	2.88	2.84	2.84
	S_3P	3.77	2.90	3.04	3.80
325 atom models	S_3O	2.84	2.86	2.81	2.78
	S_3P	3.70	2.83	2.78	3.79

Table S3. RMSDs (Å) of the 126-atom and 325-atom S_3-state models with respect to the available XFEL crystal structures (in all cases the monomer A was used).

	Model	**6JLL**		**5WS6**		**6DHO**	
		25 atoms	Mn atoms	25 atoms	Mn atoms	25 atoms	Mn atoms
126 atom models	**S_3O**	0.33	0.13	0.31	0.16	0.29	0.16
	**S_3P**	0.28	0.09	0.21	0.12	0.30	0.14
325 atom models	**S_3O**	0.26	0.12	0.28	0.15	0.25	0.16
	**S_3P**	0.28	0.13	0.23	0.13	0.29	0.14

Magnetic Properties

Electron paramagnetic resonance (EPR) studies of the S_3 state of the OEC indicate an heterogeneous mixture of at least two different forms of the complex with S = 3,[7-9] but the observed signals can be reproduced only if all four Mn ions are present in the IV oxidation state.[8] Broken-symmetry DFT calculations on the **S_3O** and **S_3P** models suggest that they both have a S = 3 ground spin state, in agreement with EPR experiments and previous computational studies with larger models. The pairwise exchange coupling constants, J_{ij}, of the four Mn ions, computed by diagonalization of the Heisenberg spin Hamiltonian, are presented in Table S4.

Table S4. Calculated single-point relative energies (cm^{-1}) of the different BS-states used to derive the exchange coupling constants, J_{ij}, of the 126-atom models.

	Spin Configuration Mn1 Mn2 Mn3 Mn4							
Model	*αααα*	*αααβ*	*ααβα*	*αβαα*	*βααα*	*ααββ*	*αβαβ*	*αββα*
**S_3O**	0	-161.6	-155.9	259.7	176.1	117.3	82.7	-86.2
**S_3P**	0	221.3	511.2	-217.8	-360.7	89.1	-28.5	91.8

Table S5. Calculated exchange coupling constants and spin of the ground (S_{GS}) and first excited (S_{EXC}) states along with their energy separation for the two S_3-state forms (126-atom models).

	Pairwise exchange coupling constants, J (cm^{-1})						Magnetic states			
Model	J_{12}	J_{13}	J_{14}	J_{23}	J_{24}	J_{34}	S_{GS}	S_{EXC}	ΔE (cm^{-1})	Spin conf.
**S_3O**	17.57	-3.57	5.48	10.44	0.74	-24.24	3	4	31.8	*αααβ*
**S_3P**	-27.87	7.44	-7.26	11.15	1.31	26.80	3	4	41.5	*βααα*

DLPNO-CCSD(T) Methodology

In the DLPNO-CCSD(T) methodology,[10-13] orbital domains are initially constructed based on the Mulliken population of each localized orbital on each atom, derived by a fast semi-canonical local MP2 calculation. Atoms with populations larger than a given threshold (T_{CutMKN}) are included in the orbital's domain. Pair correlation energies derived from a semi-canonical local MP2 (SC-LMP2) calculation are used as a criterion based on another threshold (T_{CutPairs}) to separate the pairs to ‘’strong pairs’’, which will be part of the coupled cluster iterations and ‘’weak pairs’’, whose LMP2 correlation energy will be added to the final energy. Notably, “TightPNO” settings trigger the full iterative DLPNO-MP2 treatment, whereas “LoosePNO” and “NormalPNO” options use the semicanonical LMP2 correlation energies for the weak pairs. The (SC-)LMP2 pair correlation energies of the surviving pairs confined to the corresponding pair domains are computed. The pair natural orbitals (PNOs) with pair density eigenvalues larger than the T_{CutPNO} threshold will be the final PNO basis for the coupled cluster calculations. T_{CutPNO} is the most critical parameter for the accuracy of the calculation.

Calculation of the perturbative triples correction for the DLPNO-CCSD method can be performed either using the semi-canonical approximation or iteratively. For the semi-canonical perturbative triples calculation, here denoted (T_0), a set of triples-natural orbitals (TNOs) is constructed, starting from the PNOs of both the “strong” and the “weak” pairs produced for the preceding DLPNO-CCSD calculation. The domain of each triple is constructed as the union of the individual orbital domains. The truncated TNO basis is quasi-canonicalized via the Fock operator and the integrals over TNOs are constructed. Off-diagonal contributions from the internal Fock matrix F_{ij} are neglected in the calculation semicanonical triples correction, (T_0). The more accurate iterative perturbative triples correction, here denoted (T_1), involves only the most important triples calculated in the DLPNO-CCSD(T_0) calculation, that are the triples that make up 90% of the (T_0) energy. The extension from the (T_0) to the (T_1) approach in this subset of TNOs involves iterative calculation of the triples amplitudes, starting from the (T_0) values, until convergence is achieved.

Extrapolation to the Complete Basis Set Limit

In order to approach the complete basis set (CBS) limit with respect to Mn ions, we employ a two-point extrapolation scheme.[14-15] The HF energy extrapolation is performed using the equation:

$$E_{\mathrm{HF}}^{\mathrm{CBS}} = \frac{e^{a\sqrt{X}}\, E_{\mathrm{corr}}^{(X)} - e^{a\sqrt{Y}}\, E_{\mathrm{corr}}^{(Y)}}{e^{a\sqrt{X}} - e^{a\sqrt{Y}}} \qquad \text{eq S1}$$

The CBS limit correlation energy is estimated according to the equation:

$$E_{\mathrm{corr}}^{\mathrm{CBS}} = \frac{X^{\alpha}\, E_{\mathrm{corr}}^{(X)} - Y^{\alpha}\, E_{\mathrm{corr}}^{(Y)}}{X^{\alpha} - Y^{\alpha}} \qquad \text{eq S2}$$

where $\alpha = 2.97$ and X and Y are the corresponding successive cardinal numbers of the basis set (X = 3 for TZ/TZ basis sets and Y = 4 for QZ/TZ basis sets).[16]

The term $\delta_{\mathrm{corr}}^{\mathrm{CBS}}$ was defined as:

$$\delta_{\mathrm{corr}}^{\mathrm{CBS}} = E_{\mathrm{corr}}^{\mathrm{CBS}} - E_{\mathrm{corr}}^{(X)} \qquad \text{eq S3}$$

and was used to estimate the CBS and PNO space extrapolated energies.

Assessment of Multireference Character

One way to characterize the multireference character of the wavefunction is the T_1 diagnostic.[17] A value of T_1 larger than 0.05 is suggested to indicate substantial multireference character in 3d TM species.[18] In our case, the calculated T_1 values are less than 0.02 in all DLPNO-CCSD calculations for both structures. Additionally, if the value of the largest double excitation amplitudes is significantly larger than 0.1, the system is considered to show significant non-dynamical correlation effects.[19] In our case, the largest double excitation amplitudes values are smaller than 0.08 in all DLPNO-CCSD calculations for both structures. Thus, the single-reference CCSD method is appropriate to describe the system under study. Notably, the coupled cluster expansion was performed using orbitals obtained using the B3LYP functional, thus the extrapolated "HF" energy is the energy of the reference determinant formed from the Kohn-Sham orbitals. Kohn-Sham orbitals are superior to a HF reference for CCSD(T), because they provide a reference determinant with decreased spin contamination.[20]

Detailed DLPNO-CCSD(T) Results

Table S6. Correlation Energy Contributions and Relative energies between the S_3-hydroxo (**S_3O**) and S_3-peroxo (**S_3P**) forms calculated using the DLPNO-CCSD(T) method using different levels of approximation.

	Basis set	T_{CutPNO}	$T_{CutPairs}$	T_{CutDO}		E_{corr} $^{DLPNO-CC}$, Hartree **S_3O**	**S_3P**	ΔE_{PO}, kcal mol^{-1}
LoosePNO	def2-TZVP	1.0×10^{-6}	1.0×10^{-3}	2.0×10^{-2}	CCSD[a]	-13.67088	-13.54315	80.1
					LMP2[b]	-0.89412	-0.84668	29.8
					(T_0)	-0.59343	-0.54168	32.5
					(T_1)	-0.63389	-0.57524	36.8
					E_{corr}[c]	-15.19889	-14.96506	146.7
LoosePNO	def2-TZVP Mn: def2 - QZVPP	1.0×10^{-6}	1.0×10^{-3}	2.0×10^{-2}	CCSD[a]	-14.16082	-14.02847	83.0
					LMP2[b]	-0.97414	-0.93383	25.3
					(T_0)	-0.62604	-0.57222	33.8
					(T_1)	-0.66769	-0.60697	38.1
					E_{corr}[c]	-15.80265	-15.56927	146.4
NormalPNO	def2-TZVP	1.0×10^{-6}	1.0×10^{-4}	1.0×10^{-2}	CCSD[a]	-14.03120	-13.88106	94.2
					LMP2[b]	-0.29505	-0.29173	2.1
					(T_0)	-0.66199	-0.60609	35.1
					(T_1)	-0.71521	-0.65023	40.8
					E_{corr}[c]	-15.04147	-14.82302	137.07
NormalPNO	def2-TZVP	3.33×10^{-7}	1.0×10^{-4}	1.0×10^{-2}	CCSD[a]	-14.10149	-13.95133	94.2
					LMP2[b]	-0.22469	-0.22053	2.6
					(T_0)	-0.67379	-0.61739	35.4
					(T_1)	-0.72862	-0.66297	41.2
					E_{corr}[c]	-15.05480	-14.83483	138.0
NormalPNO	def2-TZVP Mn: def2- QZVPP	3.33×10^{-7}	1.0×10^{-4}	1.0×10^{-2}	CCSD[a]	-14.62605	-14.47641	93.9
					LMP2[b]	-0.27448	-0.26873	3.6
					(T_0)	-0.70932	-0.65102	36.6
					(T_1)	-0.764529	-0.697181	42.3
					E_{corr}[c]	-15.66506	-15.44232	139.8

	def2-TZVP	1.0 × 10^{-7}	1.0 × 10^{-4}	1.0 × 10^{-2}	CCSD[a]	-14.14464	-13.99518	93.8
					LMP2[b]	-0.18353	-0.17974	2.4
					(T_0)	-0.68134	-0.62415	35.9
					(T_1)	-0.73715	-0.67056	41.8
					E_{corr}[c]	-15.06532	-14.84547	138.0
	def2-TZVP<br>Mn: def2-<br>∞<br>ZVPP	3.33 × 10^{-7}	1.0 × 10^{-4}	1.0 × 10^{-2}	CCSD[a]	-15.01462	-14.86536	93.7
					$\delta_{\mathrm{CCSD}}^{\mathrm{CBS}}$	-0.91313	-0.91403	-0.6
					LMP2[b]	-0.31135	-0.30443	4.4
					$\delta_{\mathrm{MP2}}^{\mathrm{CBS}}$	-0.08666	-0.08390	1.7
					(T_0)	-0.73564	-0.67594	37.5
					$\delta_{(\mathrm{T_0})}^{\mathrm{CBS}}$	-0.06185	-0.05855	2.1
					(T_1)	-0.79113	-0.72252	43.1
					$\delta_{(\mathrm{T_1})}^{\mathrm{CBS}}$	-0.06251	-0.05955	1.9
					E_{corr}[c]	-16.11710	-15.89231	141.1
	def2-TZVP<br>Mn: def2-<br>∞<br>ZVPP	∞<br>PNO limit[d]	1.0 × 10^{-4}	1.0 × 10^{-2}	CCSD[a]	-15.11450	-14.96627	93.0
					LMP2[b,e]	-0.27019	-0.26363	4.1
					(T_0)	-0.75287	-0.69173	38.4
					(T_1)	-0.81063	-0.68072	44.2
					E_{corr}[c]	-16.19531	-15.97017	40.2
TightPNO	def2-TZVP	1.0 × 10^{-6}	1.0 × 10^{-5}	5.0 × 10^{-3}	CCSD[a]	-14.08042	-13.93183	93.2
					LMP2[b]	-0.20951	-0.20882	0.4
					(T_0)	-0.67275	-0.61627	35.4
	def2-TZVP<br>Mn: def2-<br>QZVPP	1.0 × 10^{-6}	1.0 × 10^{-5}	5.0 × 10^{-3}	CCSD[a]	-14.57783	-14.42958	93.0
					LMP2[b]	-0.29450	-0.29223	1.4
	def2-TZVP<br>Mn: def2-<br>∞	1.0 × 10^{-6}	1.0 × 10^{-5}	5.0 × 10^{-3}	CCSD[a]	-14.94628	-14.79829	92.8
					$\delta_{\mathrm{CCSD}}^{\mathrm{CBS}}$	-0.86587	-0.86646	-0.4
					LMP2[b]	-0.35745	-0.35402	2.15
					$\delta_{\mathrm{MP2}}^{\mathrm{CBS}}$	-0.14794	-0.14520	1.7
	def2-TZVP	3.3 × 10^{-7}	1.0 × 10^{-5}	5.0 × 10^{-3}	CCSD[a]	-14.15878	-14.00966	93.6
					LMP2[b]	-0.12579	-0.12416	1.0

				CCSD[a]	-14.20681	-14.05719	93.9
def2-TZVP	1.0 × 10^{-7}	1.0 × 10^{-5}	5.0 × 10^{-3}	LMP2[b]	-0.07659	-0.07528	0.8
				(T_0)	-0.69748	-0.63959	36.3
def2-TZVP				CCSD[a]	-15.18313	-15.03390	93.6
Mn: def2-<br>∞ ZVPP	∞ PNO limit[d]	1.0 × 10^{-5}	5.0 × 10^{-3}	LMP2[b,e]	-0.07659	-0.07528	0.8
				(T_0)	-0.77170	-0.70980	38.8

[a] Only “strong” pairs are part of the coupled cluster iterations. [b] For the “weak” pairs the (SC-)LMP2 energy is used. [c] Estimated as the sum of SD, MP2 and (T_1) values. [d] PNO extrapolations were estimated according to the equation $E^\infty = E^x + F \cdot (E^y - E^x)$, where x = -log (T_{CutPNO}), y = = -log (T_{CutPNO}) + 1, and F = 1.5. [e] The LMP2 values are not extrapolated.

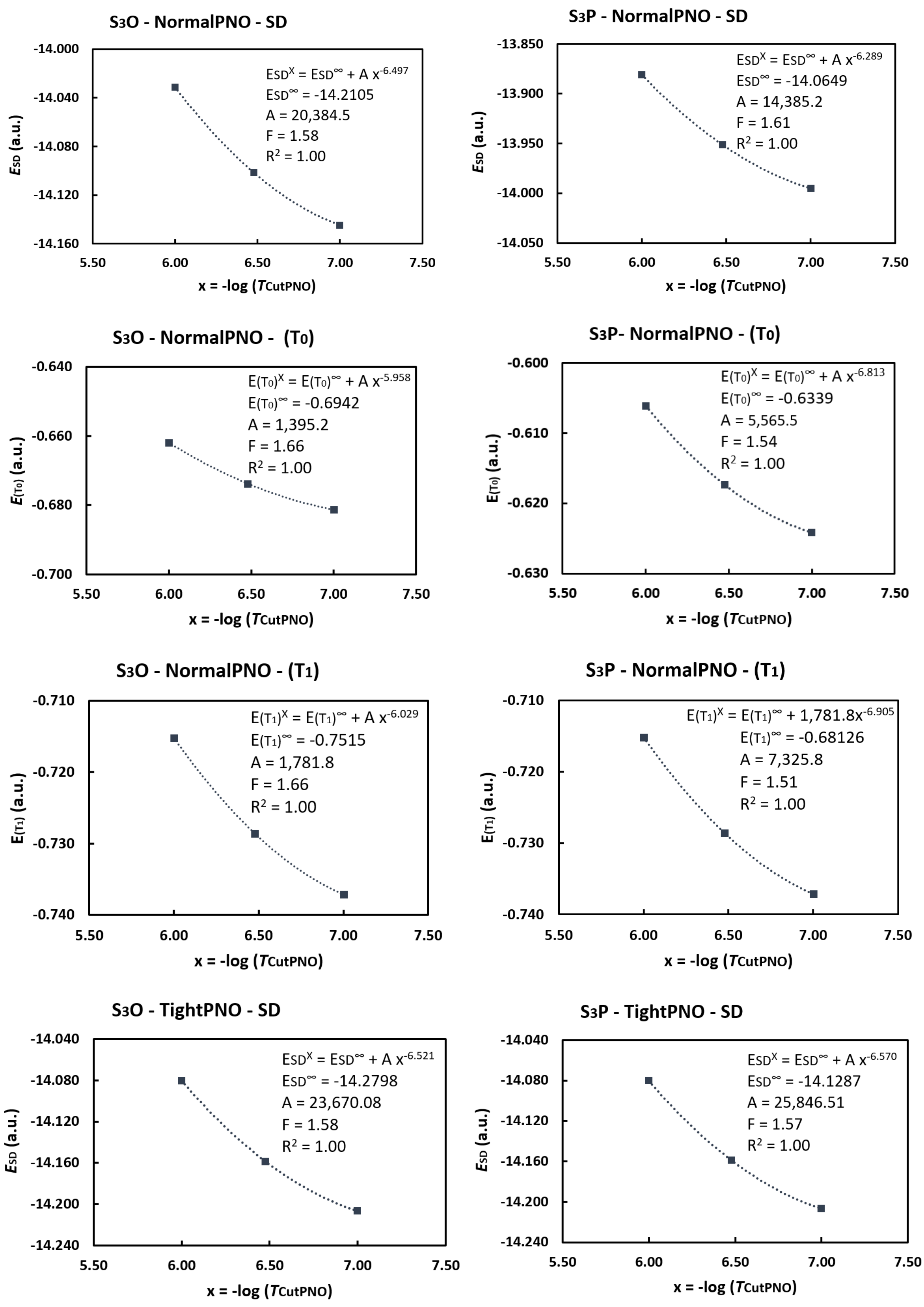

Figure S3. Dependence of the DLPNO-CCSD(T)/def2-TZVP correlation energy contributions on the exponent *x* of T_{CutPNO} threshold for **S$_3$O** and **S$_3$P**. E_{SD}^{∞}, $E_{(T0)}^{\infty}$ and $E_{(T1)}^{\infty}$ correspond to the CCSD, (T$_0$) and (T$_1$) correlation energy components at the complete PNO space limit. *F* is defined as: $F = \frac{y^{\beta}}{y^{\beta} - x^{\beta}}$, where $x = 6$ and $y = 7$.

References

[1] M. Suga, F. Akita, M. Sugahara, M. Kubo, Y. Nakajima, T. Nakane, K. Yamashita, Y. Umena, M. Nakabayashi, T. Yamane, T. Nakano, M. Suzuki, T. Masuda, S. Inoue, T. Kimura, T. Nomura, S. Yonekura, L.-J. Yu, T. Sakamoto, T. Motomura, J.-H. Chen, Y. Kato, T. Noguchi, K. Tono, Y. Joti, T. Kameshima, T. Hatsui, E. Nango, R. Tanaka, H. Naitow, Y. Matsuura, A. Yamashita, M. Yamamoto, O. Nureki, M. Yabashi, T. Ishikawa, S. Iwata, J.-R. Shen, *Nature* **2017**, *543*, 131-135.

[2] J. Kern, R. Chatterjee, I. D. Young, F. D. Fuller, L. Lassalle, M. Ibrahim, S. Gul, T. Fransson, A. S. Brewster, R. Alonso-Mori, R. Hussein, M. Zhang, L. Douthit, C. de Lichtenberg, M. H. Cheah, D. Shevela, J. Wersig, I. Seuffert, D. Sokaras, E. Pastor, C. Weninger, T. Kroll, R. G. Sierra, P. Aller, A. Butryn, A. M. Orville, M. Liang, A. Batyuk, J. E. Koglin, S. Carbajo, S. Boutet, N. W. Moriarty, J. M. Holton, H. Dobbek, P. D. Adams, U. Bergmann, N. K. Sauter, A. Zouni, J. Messinger, J. Yano, V. K. Yachandra, *Nature* **2018**, *563*, 421-425.

[3] M. Suga, F. Akita, K. Yamashita, Y. Nakajima, G. Ueno, H. Li, T. Yamane, K. Hirata, Y. Umena, S. Yonekura, L.-J. Yu, H. Murakami, T. Nomura, T. Kimura, M. Kubo, S. Baba, T. Kumasaka, K. Tono, M. Yabashi, H. Isobe, K. Yamaguchi, M. Yamamoto, H. Ago, J.-R. Shen, *Science* **2019**, *366*, 334.

[4] C. Glöckner, J. Kern, M. Broser, A. Zouni, V. Yachandra, J. Yano, *J. Biol. Chem.* **2013**, *288*, 22607-22620.

[5] M. Askerka, J. Wang, D. J. Vinyard, G. W. Brudvig, V. S. Batista, *Biochemistry* **2016**, *55*, 981-984.

[6] M. Haumann, C. Müller, P. Liebisch, L. Iuzzolino, J. Dittmer, M. Grabolle, T. Neisius, W. Meyer-Klaucke, H. Dau, *Biochemistry* **2005**, *44*, 1894-1908.

[7] N. Cox, M. Retegan, F. Neese, D. A. Pantazis, A. Boussac, W. Lubitz, *Science* **2014**, *345*, 804-808.

[8] D. A. Marchiori, R. J. Debus, R. D. Britt, *Biochemistry* **2020**, *59*, 4864-4872.

[9] M. Chrysina, E. Heyno, Y. Kutin, M. Reus, H. Nilsson, M. M. Nowaczyk, S. DeBeer, F. Neese, J. Messinger, W. Lubitz, N. Cox, *Proc. Natl. Acad. Sci. U. S. A.* **2019**, *116*, 16841.

[10] C. Riplinger, F. Neese, *J. Chem. Phys.* **2013**, *138*, 034106.

[11] C. Riplinger, B. Sandhoefer, A. Hansen, F. Neese, *J. Chem. Phys.* **2013**, *139*, 134101.

[12] M. Saitow, U. Becker, C. Riplinger, E. F. Valeev, F. Neese, *J. Chem. Phys.* **2017**, *146*, 164105.

[13] Y. Guo, C. Riplinger, U. Becker, D. G. Liakos, Y. Minenkov, L. Cavallo, F. Neese, *J. Chem. Phys.* **2018**, *148*, 011101.

[14] D. G. Truhlar, *Chem. Phys. Lett.* **1998**, *294*, 45-48.

[15] T. Helgaker, W. Klopper, H. Koch, J. Noga, *J. Chem. Phys.* **1997**, *106*, 9639-9646.

[16] F. Neese, E. F. Valeev, *J. Chem. Theory Comput.* **2011**, *7*, 33-43.

[17] T. J. Lee, P. R. Taylor, *Int. J. Quantum Chem.* **1989**, *36*, 199-207.

[18] W. Jiang, N. J. DeYonker, A. K. Wilson, *J. Chem. Theory Comput.* **2012**, *8*, 460-468.

[19] A. Beste, R. J. Bartlett, *Chem. Phys. Lett.* **2002**, *366*, 100-108.

[20] G. J. O. Beran, S. R. Gwaltney, M. Head-Gordon, *Phys. Chem. Chem. Phys.* **2003**, *5*, 2488-2493

Cartesian Coordinates of Optimized Structures

S3AW-126 atoms

Mn	24.859404863420	-35.493822186033	-61.044967288640
Mn	27.318342511345	-34.966797143311	-62.252138182576
Mn	27.376386512070	-33.030335269921	-60.159059309187
Mn	27.838927137483	-32.786763915384	-57.448899048117
Ca	27.593888598445	-36.508270855446	-59.177491118614
O	26.459037896304	-36.345154022740	-61.409340915547
O	28.303278333154	-34.464517279464	-60.800911021107
O	25.917772017892	-33.922773273977	-61.457445128285
O	28.560641481108	-32.214174177149	-59.083882137315
O	26.983422283732	-33.920818316337	-58.565657639713
O	28.859466562948	-31.194660184867	-56.474323860469
O	27.061351331079	-33.356996912403	-55.839015444715
O	28.218352506134	-38.899472366180	-59.144474660190
O	26.906074286051	-36.864479269711	-56.849354862489
H	29.404141515658	-41.150480601035	-56.339731722711
H	28.896177561129	-39.651812077001	-55.528918831477
C	28.540000236908	-40.610451498694	-55.923430965150
O	27.581554092681	-40.346057394821	-56.877600223812
H	30.721883145369	-35.082789316507	-54.974125758677
C	31.030773710740	-35.223050928749	-56.024220038022
C	29.885652010109	-34.936199285955	-56.983961409868
O	29.478509894776	-35.787647304864	-57.784513334639
O	29.410057015681	-33.730159106167	-56.883366458088
H	21.413556924547	-41.050097885261	-59.196527708688
C	22.131933348294	-41.425891134821	-59.921478468061
H	22.409347175097	-42.483280824304	-59.799132701098
C	23.384860816168	-40.547747645730	-59.858441955436
C	22.991083959612	-39.070795613526	-59.898708180593
C	24.152280777340	-38.100817449382	-59.993232652853
O	25.188906495437	-38.239690451538	-59.328787464856
O	23.916336963870	-37.131952810467	-60.830872833025
H	19.161448408483	-31.467692582775	-59.808573573786
C	20.064923981282	-31.358901219924	-59.207853565398
H	20.863804611628	-30.810094034716	-59.728046081446
C	20.567844805070	-32.716994083839	-58.689681890054
C	21.402763222557	-33.472784316646	-59.669332443465
N	20.950688119873	-33.993433012797	-60.870958053322
C	22.736517443588	-33.787325587421	-59.629625271831
C	21.978332872626	-34.595031626773	-61.503575639817
N	23.079357039552	-34.483212949877	-60.772068959364
H	22.052928883446	-29.449388276909	-59.342295777697
C	22.848974896436	-28.811512193479	-59.753908222522
H	22.549093107693	-28.354476691943	-60.702577799038
C	24.146516790072	-29.604168483328	-59.887676536368
C	24.635086605562	-30.147869391251	-58.541386377852
C	25.762433864194	-31.150325263989	-58.650993599889
O	26.000617819127	-31.654844907411	-59.783109896850
O	26.387423432941	-31.455968192431	-57.581183743307
H	24.476336191610	-30.911381289602	-66.368911027242
C	24.450296094061	-31.313554757701	-65.356153271841
N	23.447661722813	-30.976408979765	-64.468664652469
C	25.272460227356	-32.181259325128	-64.691256342419

C	23.646131790139	-31.605478917725	-63.306780911413
N	24.750315348471	-32.337874507873	-63.421840810767
H	24.752716078750	-36.896895320338	-67.326995670358
C	25.285844310565	-36.659368021310	-66.406761172165
H	25.947268116417	-37.508338749360	-66.179236990368
C	24.364456393730	-36.296335524197	-65.245104250761
C	25.031924073999	-35.769007766815	-63.976158444584
O	26.251141570529	-35.434966857073	-63.967996077389
O	24.270289757958	-35.667429156821	-62.957796348685
H	29.320056907572	-38.105510603285	-64.144751285007
C	29.989026446703	-37.970239479616	-63.286288056569
C	29.278840950714	-37.048673384980	-62.323511117337
O	29.251523015405	-37.228937199200	-61.100484571270
H	30.910268224416	-37.492080397162	-63.643991464314
O	28.686851358868	-36.063775058191	-62.947301805663
H	30.677957003039	-30.284178061805	-66.089026784506
C	30.995557935148	-30.840329103466	-65.198282056069
H	31.946759698883	-31.344513147461	-65.359249170636
C	29.995499264604	-31.869961388312	-64.656563943163
C	28.867285314011	-31.256436670623	-63.808024517237
C	28.216284453342	-32.261072594493	-62.879807301833
O	27.987594701874	-33.429398408756	-63.329538623817
O	27.927076380125	-31.884297811347	-61.704041345194
O	26.701239988543	-39.174006437684	-62.007143938832
O	25.112361719120	-38.921040497081	-56.604677672506
O	27.626881424252	-35.715037908432	-54.234170820706
H	26.182140500816	-32.958380653382	-55.751943580473
H	25.528738776307	-34.641471024423	-58.901305747283
H	29.177319465770	-30.540874335219	-57.120033832774
H	29.660001856297	-31.581120927614	-56.078864825997
H	26.880649582170	-36.300119760308	-53.930446933464
H	27.230986840587	-34.912330544940	-54.640442874560
H	26.203058040841	-37.502328267951	-56.586584526380
H	25.006182743661	-38.819332351245	-57.576008139875
H	25.872295109371	-39.529004094970	-56.526165292212
H	27.303649724153	-36.524303377517	-56.025368771421
H	28.093831311947	-39.467587592301	-58.351840467179
H	29.080894688820	-39.114167055689	-59.527850435409
H	27.249695812599	-41.181745886318	-57.241751865184
H	27.036863231537	-39.383988829226	-61.121131487533
H	26.523388707033	-38.215709183267	-61.944780431318
H	23.452261889702	-33.576446419971	-58.847499823481
H	21.899466174002	-35.106564217270	-62.454060550574
H	20.000182135959	-33.953164271585	-61.218031346174
H	25.161163742069	-32.922486861933	-62.652443400261
H	23.013749515902	-31.535907462498	-62.430943043049
H	26.166669037245	-32.700144376682	-65.010034199495
H	22.672087385444	-30.351797306872	-64.659863678841
H	31.199681534242	-30.083656836546	-64.425217652477
H	25.944140192833	-35.813163344755	-66.644680253967
H	30.232614329605	-38.927204801553	-62.817462041592
H	19.769858275410	-30.738058868240	-58.350035663628
H	22.978940193672	-27.980960614843	-59.043644093678
H	21.655907433325	-41.333656303827	-60.908788763064
H	22.436860986191	-38.808180681003	-58.982721957701
H	24.057888069135	-40.777455561063	-60.696709854466
H	23.651621305729	-35.514103949988	-65.548545740931

H	19.715477393884	-33.332246644106	-58.365820353850
H	23.819405078824	-30.672401145867	-58.017656616884
H	24.933480320647	-28.980072175524	-60.334108970910
H	31.391005671907	-36.245844676785	-56.164886702031
H	23.747826332510	-37.149791464749	-64.933171662036
H	21.192719623400	-32.565928134634	-57.800771766630
H	24.954999262011	-29.343497818012	-57.865688083762
H	23.997455937081	-30.444963102061	-60.573756276251
H	22.313009688408	-38.878597928893	-60.738454294598
H	30.551698006478	-32.572497466329	-64.020931734282
H	29.246955052414	-30.438248180243	-63.185166615833
H	29.570109533824	-32.473438962442	-65.468681325228
H	31.850103921410	-34.519656050037	-56.219858268948
H	23.948448691079	-40.754244598210	-58.938418205199
H	28.077940358401	-30.828960325686	-64.445344534311
O	25.121943218836	-35.466202125174	-59.254401593293
H	28.136965680519	-41.196064685295	-55.083470755242

S3PerOxoA-126 atoms

Mn	24.847288028593	-35.449272576882	-60.929841094428
Mn	27.262582635841	-35.001008482000	-62.293770723625
Mn	27.247005635049	-33.120533473286	-60.239824811329
Mn	27.752452794422	-32.834598879878	-57.343259865925
Ca	27.668402442435	-36.523657956371	-59.223239415623
O	26.422701042271	-36.339543223929	-61.362852010822
O	28.275317306329	-34.493803114661	-60.868508016598
O	25.939515842977	-33.858273123581	-61.530481613221
O	28.430012910662	-32.488454339399	-58.974647349429
O	26.667381537336	-34.213442618566	-58.778272493012
O	28.722590486685	-31.114112185005	-56.145737114544
O	26.746820740011	-33.473986781512	-55.640540643609
O	28.207650326157	-38.952336258158	-59.199287078502
O	27.212259034895	-36.961614957985	-56.828869096153
H	29.393555781149	-41.171909004115	-56.333156360022
H	28.913995512338	-39.650806969703	-55.548165791763
C	28.540000224644	-40.610451526985	-55.923430970985
O	27.581554124522	-40.346057351606	-56.877600214993
H	30.721883145790	-35.082789301982	-54.974125732327
C	31.026006937860	-35.267829115453	-56.014583647560
C	29.880617784759	-34.945694612100	-56.957398972843
O	29.595542640083	-35.684278321681	-57.912502056785
O	29.249294787122	-33.855080140943	-56.655168077765
H	21.413556924578	-41.050097898046	-59.196527708552
C	22.131933348705	-41.425891085113	-59.921478474545
H	22.409347175081	-42.483280844167	-59.799132683895
C	23.390578390780	-40.542037625743	-59.818023966298
C	22.994956747050	-39.077224165716	-59.582036262326
C	24.121130632087	-38.059008080395	-59.692200387014
O	25.181043137238	-38.173684198431	-59.039823174036
O	23.857848851277	-37.082646022587	-60.487404719900
H	19.161448408116	-31.467692577179	-59.808573596589
C	20.064923976265	-31.358901237038	-59.207853525697
H	20.863804640161	-30.810094006666	-59.728046104094
C	20.543334404829	-32.688197382930	-58.608228126886
C	21.396033027523	-33.475703964100	-59.538591340464
N	20.963807946078	-33.982136139802	-60.751706901154

C	22.730087121968	-33.786436518978	-59.475863276504
C	22.003187344877	-34.571729612677	-61.374416592825
N	23.090317175645	-34.462770384590	-60.623132500977
H	22.052928846947	-29.449388288166	-59.342295820408
C	22.848974980927	-28.811512207367	-59.753908106961
H	22.549093059964	-28.354476684629	-60.702577864747
C	24.138219274391	-29.610026414542	-59.884548188576
C	24.588900547380	-30.174152276902	-58.537200560810
C	25.687240107546	-31.206639440999	-58.634044977679
O	25.940774350015	-31.689544117906	-59.771653618394
O	26.265688334619	-31.548130145873	-57.549155880111
H	24.476336189888	-30.911381284703	-66.368911056385
C	24.450296090812	-31.313554768462	-65.356153236014
N	23.446025752117	-30.988890273724	-64.466643441588
C	25.280641944324	-32.176996349704	-64.696069046754
C	23.655008640084	-31.623065995466	-63.307541210213
N	24.764297982670	-32.344591442517	-63.427423887697
H	24.752716072220	-36.896895317837	-67.326995666232
C	25.285844329055	-36.659368026988	-66.406761170105
H	25.947268110387	-37.508338740040	-66.179236991223
C	24.326213641788	-36.324619877435	-65.274818147275
C	24.932992833010	-35.785326884946	-63.983375223164
O	26.167254218945	-35.449999147020	-63.960008747157
O	24.152639188282	-35.674820452483	-63.002645517157
H	29.320056907905	-38.105510603091	-64.144751286152
C	29.994431165592	-37.978954495302	-63.292772816884
C	29.266144383858	-37.064935052243	-62.338783951342
O	29.271387351590	-37.225819476635	-61.110619025072
H	30.913005700921	-37.493862215911	-63.647794674672
O	28.625428229421	-36.126443285289	-62.975887675872
H	30.677956990573	-30.284178061493	-66.089026762894
C	30.995557982743	-30.840329104455	-65.198282094038
H	31.946759674441	-31.344513141457	-65.359249153592
C	29.996460092387	-31.872743193587	-64.660435207249
C	28.878625365030	-31.265327554959	-63.796785081328
C	28.216760931349	-32.279864314121	-62.883851142874
O	27.977121576417	-33.436286290157	-63.356822705342
O	27.933588529543	-31.907658985611	-61.702553079677
O	26.612175784363	-39.132429065023	-61.903411690818
O	25.244850489414	-38.810643824174	-56.374777191282
O	27.626881504210	-35.715037905117	-54.234171351805
H	26.275238001735	-32.830894710282	-55.090381481542
H	27.131313294319	-34.163687630736	-55.047809114184
H	28.629819794052	-30.235892207376	-56.545938116257
H	29.677072949630	-31.250392239612	-56.040809356892
H	26.880649446397	-36.300119804051	-53.930446455502
H	28.323819338968	-35.816804716225	-53.568179603896
H	26.468041243700	-37.536213358756	-56.529840563940
H	25.063945034694	-38.692483222147	-57.337499061151
H	25.951691288384	-39.484193491240	-56.371809558858
H	27.568293300725	-36.541825658471	-56.027968114554
H	28.081518885954	-39.489752566394	-58.386102907477
H	29.069270224711	-39.186997961820	-59.573348994480
H	27.230241900274	-41.180774492176	-57.225249956262
H	26.993288212772	-39.334405741875	-61.033353205266
H	26.458660140833	-38.166065199609	-61.851467685414
H	23.448811480252	-33.594378314380	-58.690034891390

H	21.948108999411	-35.070475198884	-62.333203658562
H	20.020989973195	-33.930424252219	-61.116921707349
H	25.197070692682	-32.934593671133	-62.656472327957
H	23.023255046943	-31.562996803084	-62.430641575653
H	26.178651879303	-32.687571833379	-65.017285825037
H	22.664292775014	-30.371348210383	-64.653653077417
H	31.198352247317	-30.084515881129	-64.424065926669
H	25.935741605873	-35.803276350180	-66.628979094017
H	30.242368374492	-38.936889648436	-62.827941592947
H	19.756185139794	-30.701609634037	-58.380342269731
H	22.979746300311	-27.981673508311	-59.042773785843
H	21.667371065812	-41.316290581299	-60.911475710497
H	22.567463588009	-38.978799778261	-58.571491142309
H	23.997011451210	-40.627781303905	-60.730591379122
H	23.603720632131	-35.560863640367	-65.601576373100
H	19.676859778305	-33.281592080870	-58.280927573456
H	23.748926637456	-30.677471155644	-58.031400408084
H	24.939374585841	-28.988568052950	-60.309062604437
H	31.355591103262	-36.303122889961	-56.138818143921
H	23.720855207000	-37.195521880808	-64.989296291615
H	21.144445786735	-32.497057899960	-57.710877052978
H	24.918284280903	-29.382344293695	-57.850931976882
H	23.992029822991	-30.439969651004	-60.583988070099
H	22.209486143146	-38.784765122990	-60.288485682850
H	30.554434967602	-32.584845013213	-64.036910615528
H	29.270697240991	-30.462520683246	-63.161429568171
H	29.563339315959	-32.465852195745	-65.476157079989
H	31.867674752056	-34.598072348798	-56.237283325286
H	24.025853422067	-40.883503168342	-58.989014743398
H	28.091950767022	-30.816608639640	-64.422968014935
O	25.500524639932	-35.030267086899	-58.929527791998
H	28.131693784367	-41.174316425647	-55.071030880766

S3AW-325 atoms

Mn	24.860498917873	-35.541499563761	-61.087082244828
Mn	27.297461592144	-35.017430689647	-62.298080671254
Mn	27.384651638040	-33.094285093216	-60.224992570140
Mn	27.680279131733	-32.866564873474	-57.449102681969
Ca	27.629672556008	-36.457440696466	-59.263984154563
O	26.459152681084	-36.407898833705	-61.463034487495
O	28.306850561250	-34.543044419297	-60.865540099886
O	25.935002427735	-33.969181378890	-61.510240703121
O	28.582174660375	-32.372516183420	-59.087944776644
O	26.910148384522	-33.960676371996	-58.687468507618
O	28.491735506451	-31.489120218604	-56.266044210527
O	26.605907143784	-33.436848416875	-56.104865091367
O	28.032184760292	-38.880480128562	-59.145044179001
O	27.224584191697	-36.775022854682	-56.901834244460
O	25.117024696178	-35.504974127634	-59.323648454575
H	25.977416001965	-32.744008628317	-55.806996921172
H	25.559628242391	-34.679457572682	-58.984015253197
H	28.510473083030	-30.523160958370	-56.575526758099
H	29.269186851185	-31.714345680233	-55.710155314009
H	26.535463989385	-37.434171029430	-56.671778588209
H	27.512647570998	-36.395012697153	-56.031463358980
H	27.873080214231	-39.379921727192	-58.315614184008

H	28.814318086961	-39.316297198469	-59.559929458084
H	30.259117305469	-26.572075683538	-57.245705384460
C	30.208668359046	-27.062952583902	-58.211858205272
H	30.809333622172	-26.572851077566	-58.990327000142
C	28.759633723157	-27.249737789018	-58.647082057847
C	28.029366681069	-28.198208897879	-57.708124509448
O	28.722281008572	-29.137928007625	-57.209006715276
O	26.800361941272	-28.019517272576	-57.471235924698
H	33.579853382753	-42.125884142513	-52.819224494696
C	32.861365410428	-41.766294443528	-53.560685583829
H	33.295400311702	-40.920562630658	-54.120547430594
C	31.546389499857	-41.405792419868	-52.854769444592
C	30.427894002418	-41.154776453659	-53.831544182206
C	29.877084690043	-42.210331416193	-54.565038855506
C	29.955514772324	-39.866913758601	-54.084572741633
C	28.917124447581	-41.989720013669	-55.545054771275
C	28.988946911233	-39.627189725488	-55.056582672566
C	28.485440373647	-40.685439778027	-55.809634279783
O	27.589885356081	-40.415853884680	-56.812228571682
H	31.915302044990	-39.374009710966	-58.358152852022
C	31.217176487539	-40.146840222397	-58.004099539531
C	30.693552933909	-40.970416082859	-59.157568070541
O	30.019513197100	-40.463952879321	-60.077911312620
N	30.951060346163	-42.283811388365	-59.142770506358
H	33.829313276674	-32.277305511279	-54.652832451168
C	32.918030746350	-31.686518313249	-54.663245775987
C	31.672801792706	-32.531998939693	-54.620118726387
O	30.549240717506	-32.032330818718	-54.437866561215
C	32.888646574368	-30.739163323801	-55.871815888294
O	32.833961197109	-31.473579333752	-57.094988896536
N	31.864669145814	-33.857514888019	-54.778434461777
C	30.807609983577	-34.845587457864	-54.688574774488
C	30.964793239229	-35.728199815533	-53.432169872100
O	30.233956994378	-36.712584805607	-53.261834341839
C	30.657085692124	-35.653223471364	-55.984857849415
C	29.773860622714	-35.026046328609	-57.070846748616
O	29.722038677176	-35.595515256034	-58.184925799893
O	29.114114533697	-33.985634014515	-56.736638768790
N	31.869980486762	-35.348622649216	-52.511084498478
C	31.875685466953	-35.915339810550	-51.169743760366
C	30.767191690363	-35.312498642196	-50.307657465340
O	30.392017454742	-34.142005496623	-50.449453899850
N	30.279070057126	-36.126702673814	-49.366189686317
H	21.660793671086	-32.991105908617	-50.393051459832
C	22.260190434514	-32.071932405647	-50.469497858292
C	22.538010407835	-31.683064799526	-51.923106425922
O	23.671137510904	-31.750528952798	-52.397735904500
N	21.483403899265	-31.262188030741	-52.640101445987
H	20.968598883031	-36.865830454628	-54.461090506770
C	21.486523238255	-36.939740890271	-55.415365488024
H	21.816273485031	-37.976748583631	-55.576238030540
C	22.630743179775	-35.926697054649	-55.403457810212
C	23.297376787519	-35.832247080223	-56.768217080686
C	22.113422563792	-34.558121573423	-54.950755675270
H	21.413557268133	-41.050099167127	-59.196526729428
C	22.131964638524	-41.425890628028	-59.921427638307
C	22.445693593914	-42.897445669032	-59.807020237346

O	22.967904863854	-43.496767136159	-60.760962440691
C	23.392005343716	-40.553835207814	-59.802556567080
C	22.966561292213	-39.090034316400	-59.915605262850
C	24.122633260880	-38.112371208937	-59.969786852348
O	25.119058811515	-38.229748659700	-59.248339024859
O	23.920203894646	-37.172994749220	-60.854556713778
N	22.168417378705	-43.521101424316	-58.641547652192
C	22.633905267262	-44.871407069461	-58.353774285047
H	21.819408103820	-45.511334034187	-58.038475003798
C	23.693482797934	-44.912037262568	-57.236166056943
C	24.903341553602	-44.104046513500	-57.539701290618
N	25.814587587785	-44.436549167130	-58.520788377595
C	25.330656951787	-42.903355888160	-57.042123559959
C	26.736578642642	-43.458417801369	-58.588153266553
N	26.472089000442	-42.509658564090	-57.703832157000
C	23.570166854703	-48.236730783898	-60.191901108607
C	24.541107810929	-47.108168087477	-60.542710867433
O	25.473969963606	-46.821416789962	-59.767047346211
H	19.161448928594	-31.467681713336	-59.808576543207
C	20.064952149632	-31.358929911597	-59.207790460249
C	21.068503419348	-30.517778282313	-59.981541584791
O	21.231233778809	-30.641049200581	-61.207642831607
C	20.623610929874	-32.729594616935	-58.762734967598
C	21.447259343434	-33.467310212993	-59.759219074164
N	20.976761510027	-34.050665770471	-60.923252998019
C	22.777731652588	-33.767364034977	-59.728738789463
C	21.995374503113	-34.668373344142	-61.547253748063
N	23.109513235046	-34.513366556810	-60.841273594027
N	21.763935066715	-29.638060576726	-59.234712966145
C	22.848989307384	-28.811527658605	-59.753880762925
H	22.549093028576	-28.354476081610	-60.702577661712
C	24.150593004573	-29.587550064062	-59.966576889937
C	24.654027789547	-30.190083468558	-58.665379437946
C	25.780175441456	-31.174420489931	-58.808071629383
O	26.042711619972	-31.673150978620	-59.939760126009
O	26.376419794452	-31.469906005906	-57.726007580453
H	25.304494141322	-28.881268394559	-66.043046569102
C	25.403148419536	-29.532130678543	-66.904494344342
H	25.187020435902	-29.020887595471	-67.847248616059
C	24.422946392715	-30.720239613363	-66.763009670937
C	24.357347430541	-31.285964184064	-65.379836286413
N	23.367655695704	-30.925100665311	-64.476565442208
C	25.158111471199	-32.156557014139	-64.697326475711
C	23.549341520960	-31.543235565352	-63.306121519976
N	24.640210545158	-32.294801452101	-63.423299216604
H	24.752715531907	-36.896895056457	-67.326995521891
C	25.285816155029	-36.659373337446	-66.406744189218
C	26.163085045034	-37.875259555086	-66.131703518409
O	25.730991908943	-38.885703129296	-65.567743271034
C	24.345719380475	-36.325757765517	-65.259342589362
C	25.020959069572	-35.804435014993	-63.997092428327
O	26.244400673764	-35.477827972655	-64.001988620380
O	24.264238290077	-35.713217907578	-62.982352657719
N	27.438237829048	-37.814819179542	-66.593279692052
C	28.315207449546	-38.972439399272	-66.569320713384
C	29.232488894332	-39.132420564725	-65.350763712320
O	29.795815522424	-40.209212307828	-65.146747257049

H	28.969452515347	-38.965885090877	-67.443621183643
N	29.396470881879	-38.034849273721	-64.594009006798
C	30.035442360362	-38.040388635491	-63.288929710789
C	29.284265408795	-37.091485629060	-62.358032922887
O	29.307429727338	-37.243470637947	-61.136205655016
H	31.079204442329	-37.709815164325	-63.348894033242
O	28.659426087213	-36.154512903514	-63.005302256570
H	29.526309006837	-30.113332414602	-66.268252553237
C	30.249626909805	-30.875810654320	-65.963876333998
H	30.684126100356	-31.307936189899	-66.868661654034
C	29.583388897463	-31.956301246124	-65.110316590953
C	28.972828017521	-31.370715236090	-63.837597449576
C	28.305751086255	-32.368840402168	-62.916493535628
O	28.031178214469	-33.524911422262	-63.371421682044
O	28.032447467051	-31.976524774700	-61.743235309873
H	36.281377094090	-31.594616742918	-63.692786901571
C	36.822737560526	-32.178259430826	-62.934187872382
H	37.552341085939	-32.868361877720	-63.352430641161
C	35.835635370410	-32.945854816721	-62.047366777862
C	35.097799057014	-34.050185349879	-62.810998848599
C	34.046311957939	-34.783933009295	-61.980881759828
N	32.958854525194	-33.860684143731	-61.638498668795
C	31.997973526230	-34.082196506689	-60.735627598687
N	31.900753115882	-35.264189602024	-60.120317733703
N	31.160397528901	-33.095373964354	-60.416427602731
H	20.765532693460	-27.013382947473	-52.914340932676
C	21.484408416322	-26.288277090069	-53.323534289478
C	22.888184006308	-26.451934624052	-52.751829180060
C	23.559893456677	-27.758845734424	-53.141884887392
N	23.651576128596	-27.917758792671	-54.635706281144
Cl	21.807421298546	-30.386615117441	-55.899426775403
O	30.650349384697	-30.603313112136	-58.567222806666
O	25.319496361270	-25.809585534134	-58.009756765196
O	26.594263500140	-39.225175648412	-61.759737785405
O	25.405823837901	-38.832492811306	-56.432816832889
O	23.900722692116	-42.726736107660	-63.250151244194
O	28.661931593683	-32.552373177613	-52.073580388639
O	24.695532608403	-31.548581183036	-54.985271011400
O	28.108097854011	-41.809292760894	-61.924124926048
O	25.978050987161	-33.364351237184	-51.435956490490
O	27.846545329297	-35.993909209152	-54.375925878100
O	24.423258801398	-40.018067823311	-63.353427306178
O	26.100549434068	-44.242507973929	-62.700129056048
O	25.754507529025	-37.545960534519	-53.856220438979
O	26.040959283281	-29.109017999111	-55.074977244729
O	23.366044343985	-25.557436377580	-56.305295602970
H	27.055980139484	-36.420784883261	-53.981347367530
H	28.618870258432	-36.412305960739	-53.942546674859
H	26.830604478620	-33.023620493790	-51.772636585563
H	29.272802751529	-33.097705827696	-51.541707044688
H	25.089205146713	-38.725834218512	-57.354058779096
H	26.142954452131	-39.485399532938	-56.501517400558
H	25.591982163851	-37.955409895427	-54.730048153318
H	24.916177322781	-37.629886639175	-53.411110279586
H	25.295161445794	-32.745047011347	-51.747756195215
H	23.882186027450	-31.293989557480	-55.468357592767
H	24.371004267324	-31.666823686479	-54.063057934583

H	27.195203191169	-41.310870206319	-57.230523477420
H	25.131587167903	-39.803102915509	-62.707924401285
H	26.748098744826	-39.320205284674	-60.800789215811
H	26.547316995609	-38.250967786303	-61.871382422804
H	24.074556704247	-41.756861225487	-63.308170860942
H	23.477084374019	-42.872073878120	-62.375123346142
H	24.758588720030	-39.648450453669	-64.193112269825
H	26.358436501311	-28.857007530955	-55.977199461568
H	25.742200248386	-30.045610670484	-55.118508590061
H	24.587564396001	-28.352118745575	-54.877273183684
H	22.924651838191	-28.555126079287	-55.000469586389
H	23.567852846563	-27.012919846765	-55.143301745299
H	21.585318511025	-31.021623584844	-53.627385189437
H	20.560437728069	-31.234848539441	-52.234565656349
H	29.144363377803	-32.369731306363	-52.897097984563
H	28.804959794887	-41.472868943855	-61.328670977840
H	27.598917543155	-41.010389216785	-62.143696808623
H	29.965135237553	-31.265892133831	-58.813386859730
H	32.042303733508	-31.178363954342	-57.604855480775
H	30.113474123161	-29.935822358708	-58.083191580014
H	26.772008775877	-43.654713195285	-62.317677854154
H	25.361764558753	-43.636401400527	-62.965965152379
H	31.511948585156	-42.709469440043	-58.420684022923
H	30.622938510367	-42.867985982265	-59.899576390730
H	24.189744464634	-25.611165130395	-56.860485314000
H	23.387391941084	-24.689958247888	-55.880933887225
H	24.681373859606	-25.738584174696	-58.763320554381
H	25.798658694687	-26.677272908796	-58.031961314692
H	32.331373800043	-36.078162389071	-60.531285307355
H	31.326432742066	-32.157883977066	-60.747845208814
H	31.140690871785	-35.425376621909	-59.456537418373
H	30.292494518609	-33.259021736038	-59.911161883456
H	32.908292074295	-32.995139460996	-62.157113015511
H	23.501463649126	-33.525236301340	-58.972473265645
H	21.904849375494	-35.217481652651	-62.468034013231
H	20.025188273823	-34.014557054148	-61.263978116073
H	25.051572606025	-32.884379727655	-62.670727968388
H	22.907906035648	-31.437134331388	-62.441770611197
H	26.039243282393	-32.690290540130	-65.006872570156
H	22.599668922457	-30.293534491105	-64.669800924802
H	27.566476683656	-43.468153924773	-59.275722242044
H	25.780520950818	-45.294382082882	-59.100824927380
H	24.895662834970	-42.301666329976	-56.259606288447
H	32.796308354957	-34.174756344577	-55.014545481147
H	21.646074461385	-29.682364560225	-58.222372325566
H	21.774367778715	-42.973271804199	-57.888797920780
H	28.825217864709	-37.221896519720	-64.793255590131
H	27.734411750026	-36.989091290425	-67.094661210733
H	37.379673384741	-31.456705339110	-62.327960344049
H	31.050349253515	-30.375248596198	-65.411465693999
H	30.673576592191	-28.039105696106	-58.073896868631
H	23.378229792199	-36.260474824826	-54.674841097546
H	25.918999445250	-35.798117631020	-66.634823525326
H	27.705418993931	-39.874675193404	-66.613389062967
H	20.766073975749	-36.691172055752	-56.203304252153
H	30.022407036737	-39.047512680992	-62.874443423386
H	19.768672545191	-30.829994076894	-58.298731815701

H	23.000657234781	-28.005551209868	-59.033052358230
H	23.035838803705	-45.256603790446	-59.287491898819
H	32.916649997918	-31.072360099225	-53.756648248071
H	32.697655025234	-42.581210556258	-54.271736756340
H	21.695831362575	-41.276531655471	-60.912877087084
H	29.880028680370	-34.291699384581	-54.521429576064
H	22.358155716411	-38.813117880178	-59.045834440459
H	24.097745058136	-40.808605873743	-60.594822820627
H	23.630540645762	-35.558816986604	-65.571776562526
H	19.783579459495	-33.350681192468	-58.437136458145
H	23.850973073032	-30.744094939729	-58.164897095877
H	24.898246402129	-28.906578674388	-60.384119801216
H	30.194524856456	-36.616833830552	-55.758036187886
H	32.024011944075	-30.075537254085	-55.783679441410
H	28.218606287691	-26.303042243840	-58.698113961849
H	28.636086438131	-38.621221373232	-55.248960460701
H	30.347602487417	-39.026737347210	-53.522946880773
H	30.211487517051	-43.226920394421	-54.377631044317
H	28.515033949030	-42.819665262745	-56.112674072085
H	31.691448376245	-40.523981632278	-52.224067214134
H	23.956356244252	-45.961380747866	-57.069641292162
H	23.759678723634	-37.198085812338	-64.963660164837
H	21.258101531855	-32.570283898896	-57.889376069733
H	24.985155934433	-29.421272134890	-57.966695461426
H	23.982776771752	-30.374465258567	-60.700772478784
H	28.721034558145	-27.698502684597	-59.646996451356
H	22.347549011140	-38.941645229203	-60.801794334914
H	30.317868731027	-32.720934389175	-64.839770234626
H	29.724931355587	-30.842154707699	-63.244840191710
H	28.805654999674	-32.461104052426	-65.689410702782
H	34.488260362141	-35.163282085620	-61.054572337291
H	34.616481574566	-33.634196665755	-63.704703197818
H	36.371107588506	-33.398514423928	-61.205403953951
H	35.816842789530	-34.795013134792	-63.164660172198
H	33.646724689496	-35.632972930452	-62.545724089523
H	33.795055560525	-30.124731812597	-55.852827643523
H	31.721269458477	-40.759567308084	-57.257212335318
H	30.379169761810	-39.634870520092	-57.532027192742
H	26.434721793219	-29.890503752603	-66.932995491458
H	24.699476376077	-31.517015860295	-67.456678598125
H	31.633256976961	-35.870214284391	-56.426301596436
H	23.416111130021	-30.396127789157	-67.039545068646
H	23.254078546512	-44.539049567405	-56.307524562569
H	32.365908207631	-34.480785521135	-52.657440784458
H	31.776035259296	-36.998532188701	-51.235703112959
H	32.837024549141	-35.680091036721	-50.709424655768
H	23.210362004013	-32.229434199149	-49.962925890927
H	21.706652701422	-31.275675591825	-49.966021547323
H	21.712849043229	-34.600833370683	-53.933204886480
H	23.653458169468	-36.806442641918	-57.105996163314
H	21.310335379209	-34.214170009736	-55.612249989116
H	24.150632542000	-35.151900469033	-56.752986740380
H	22.908113016064	-33.809086881007	-54.975015524607
H	22.586768729093	-35.464350844084	-57.514321583632
H	23.013190885798	-28.620580293403	-52.760235831598
H	22.861755145942	-26.405886560399	-51.659304061337
H	21.475290795744	-26.385831269777	-54.411899310129

H	24.578041733760	-27.800004549444	-52.755527943323
H	23.518037148992	-25.620083246690	-53.084823804328
H	21.098704491042	-25.292099472725	-53.089624304604
C	29.261833409642	-35.684476936816	-48.427545162473
H	29.616890586994	-34.822622844373	-47.859527858921
H	30.590513503638	-37.086504344935	-49.332942637602
H	29.047026379219	-36.503707963114	-47.744156734602
H	28.347609324993	-35.403363078726	-48.954722816279
H	31.275585322038	-42.232734268099	-52.189254488473
H	35.115180441342	-32.243228546817	-61.615323043419
H	23.888322717578	-40.729975170131	-58.845861833075
H	28.209202544203	-30.623990992868	-64.087469480416
H	24.137067525272	-49.168513966786	-60.133368898955
N	24.354972407231	-46.510173460572	-61.711340150615
H	23.142587283550	-48.043836464015	-59.206784949630
H	22.770462843263	-48.346686745477	-60.924460074574
H	24.975983271952	-45.749303444444	-62.005409046007
H	23.571085627131	-46.745980077931	-62.299676435941

S3PerOxoA-325 atoms

Mn	24.856441036397	-35.479116117891	-61.036360248856
Mn	27.335318910671	-35.012715413889	-62.353034471738
Mn	27.315433211844	-33.159685102264	-60.355339779115
Mn	27.633330189690	-32.961006037703	-57.404332865260
Ca	27.684259246295	-36.513471564424	-59.317797630587
O	26.459312207922	-36.335309182397	-61.436098789227
O	28.353549478294	-34.556520655830	-60.926642835540
O	26.098755806898	-33.802689855991	-61.668830493646
O	28.473618191367	-32.623608593299	-58.994905070962
O	26.606188307852	-34.252978864890	-59.012732169219
O	28.404731449721	-31.472453278722	-56.010813627377
O	26.424580476402	-33.575185283520	-55.909297745356
O	28.042821035670	-38.915693526182	-59.125665250111
O	27.242333152699	-36.758481489913	-56.916213401898
O	25.375042390028	-34.990801836375	-59.229105572026
H	25.831531056228	-32.887402024237	-55.498943689691
H	26.807891809582	-34.166227088706	-55.208904673341
H	28.441533936893	-30.536372146071	-56.352208025466
H	29.200790664767	-31.640611287310	-55.469199676806
H	26.581621892310	-37.443782777481	-56.669038494177
H	27.391480548336	-36.252192271881	-56.088346175614
H	27.874479674970	-39.407225796291	-58.293913620252
H	28.816344639831	-39.365021211174	-59.544358392689
H	30.259117307740	-26.572075693743	-57.245705390275
C	30.208668360480	-27.062952564686	-58.211858175799
H	30.809333624401	-26.572851082553	-58.990327011768
C	28.755976773172	-27.252308447505	-58.640492108206
C	28.017437042871	-28.189379093941	-57.692018050635
O	28.685242611853	-29.135237219532	-57.178842215025
O	26.785774842565	-27.994926948033	-57.462164515091
H	33.579853440840	-42.125884125213	-52.819224467833
C	32.861365302185	-41.766294480991	-53.560685641782
H	33.295400360471	-40.920562614276	-54.120547419525
C	31.563733491468	-41.376082524619	-52.836082658390
C	30.436739469122	-41.119751031359	-53.799937226941
C	29.892052388939	-42.180607976879	-54.530364492866

C	29.952724286057	-39.835262796418	-54.050874767445
C	28.930148423158	-41.970459179835	-55.508446730799
C	28.981292803587	-39.605658300272	-55.021356416762
C	28.489318846654	-40.670190886595	-55.774785968277
O	27.591564121954	-40.414042335296	-56.776741793008
H	31.915302036340	-39.374009738873	-58.358152840191
C	31.217176499731	-40.146840199510	-58.004099559728
C	30.694519438980	-40.971254141305	-59.157931893257
O	30.020268256004	-40.466861890220	-60.079299601875
N	30.951060358024	-42.283811398828	-59.142770507102
H	33.829313252620	-32.277305484117	-54.652832438798
C	32.918030771406	-31.686518330786	-54.663245795247
C	31.676268096343	-32.525527179114	-54.544268448751
O	30.577746683103	-32.023741354757	-54.250426494507
C	32.848516586145	-30.785321595124	-55.907570360479
O	32.730689107416	-31.558385755048	-57.100655832467
N	31.827771944286	-33.846792743600	-54.780428959445
C	30.734973955105	-34.790439131469	-54.656734822320
C	30.763460339345	-35.567565361532	-53.331610566792
O	29.800753843893	-36.288467156981	-53.033071991736
C	30.613602013869	-35.703302705499	-55.884855239936
C	29.744282455593	-35.136978014322	-57.008664431547
O	29.768949133991	-35.683395430936	-58.133356044797
O	28.979886845548	-34.168944593235	-56.675208906609
N	31.824391835980	-35.391681120985	-52.526453795909
C	31.875685466484	-35.915339805780	-51.169743766588
C	30.793823324758	-35.295939143023	-50.285484459262
O	30.463575153396	-34.108100958965	-50.389882811038
N	30.279011794692	-36.121565642840	-49.368274449346
H	21.660793787024	-32.991105882712	-50.393051412460
C	22.260190237182	-32.071932611946	-50.469497951658
C	22.538010588455	-31.683064257350	-51.923106316328
O	23.671137391047	-31.750529306380	-52.397736009640
N	21.485019779815	-31.263778167345	-52.641502803412
H	20.968598873892	-36.865830474093	-54.461090472803
C	21.486523260608	-36.939740872902	-55.415365531030
H	21.816273476755	-37.976748582338	-55.576238018468
C	22.621490462888	-35.917468406890	-55.414463254361
C	23.298061619156	-35.845692324690	-56.776170904649
C	22.079448704195	-34.548091452638	-54.993355769448
H	21.413557266803	-41.050099153054	-59.196526752161
C	22.131964642943	-41.425890640105	-59.921427607424
C	22.399798425617	-42.908561432752	-59.830853276582
O	22.848728025817	-43.526234653687	-60.809928783295
C	23.417010151379	-40.586512704186	-59.735875495744
C	23.000169013375	-39.129162974852	-59.484943747812
C	24.111293777089	-38.105405676459	-59.658948583960
O	25.160210615521	-38.167617806287	-58.969419791262
O	23.872098119673	-37.211390108945	-60.537985993042
N	22.173619642396	-43.518363726060	-58.644855855816
C	22.633905300475	-44.871407097256	-58.353774332552
H	21.819408082548	-45.511334014062	-58.038474973790
C	23.687833002651	-44.911872492891	-57.231563657570
C	24.900800231810	-44.104156008165	-57.523658954000
N	25.811870045181	-44.425893581903	-58.508589469247
C	25.335139528947	-42.915244030876	-57.004281049876
C	26.741401409314	-43.453349326737	-58.556187999262

N	26.481531380517	-42.518400293952	-57.655679971352
C	23.570166853424	-48.236730863552	-60.191901121479
C	24.541107730730	-47.108167864825	-60.542710823092
O	25.469472464755	-46.815021200855	-59.763822863285
H	19.161448956432	-31.467681726861	-59.808576552137
C	20.064952114940	-31.358929886044	-59.207790436432
C	21.077258511123	-30.529322150059	-59.983115166372
O	21.249994396702	-30.662376970565	-61.206091358160
C	20.597023588090	-32.722509387901	-58.719804458115
C	21.423876188749	-33.480266808987	-59.693169022549
N	20.957372567403	-34.077539054953	-60.850900568454
C	22.759059800610	-33.756175973106	-59.664767513716
C	21.983599380397	-34.683267488740	-61.474051926812
N	23.097217872069	-34.503431999151	-60.773357057477
N	21.760326636081	-29.636578353932	-59.237833657908
C	22.848989311568	-28.811527676318	-59.753880802444
H	22.549093032277	-28.354476068906	-60.702577647718
C	24.143312260624	-29.600010105358	-59.976161209624
C	24.648399684732	-30.219057449188	-58.683540552853
C	25.731978542121	-31.252450417927	-58.830993836517
O	26.054721158899	-31.671603005009	-59.980249030144
O	26.236023444988	-31.660264798691	-57.737074721864
H	25.304494127928	-28.881268380749	-66.043046578953
C	25.403148443085	-29.532130715003	-66.904494323292
H	25.187020429652	-29.020887581397	-67.847248619310
C	24.410314881515	-30.708830574134	-66.778301572937
C	24.369923681941	-31.295340300302	-65.406404522942
N	23.370415068601	-30.991114859143	-64.494939855461
C	25.226785919936	-32.117055943156	-64.732658498435
C	23.603025680058	-31.598113740223	-63.326274387903
N	24.733064728580	-32.284498712681	-63.455370935449
H	24.752715506373	-36.896895104136	-67.326995517278
C	25.285816181264	-36.659373262596	-66.406744205937
C	26.167541128177	-37.871618887868	-66.128144814613
O	25.736245482711	-38.884533931655	-65.567549307399
C	24.340826487705	-36.332423765939	-65.260262029068
C	25.004289424986	-35.803602683604	-63.992240103719
O	26.226722611623	-35.451563066732	-64.016160759836
O	24.252653326361	-35.728884613438	-62.978120993320
N	27.447435164912	-37.807593409507	-66.579611186560
C	28.315207436231	-38.972439412697	-66.569320690664
C	29.230565546498	-39.165708046558	-65.355805570184
O	29.751040277591	-40.263542611884	-65.149581340322
H	28.969452528731	-38.965885081199	-67.443621194236
N	29.444926560517	-38.070120766528	-64.609274126647
C	30.050625055847	-38.098918791841	-63.289407517257
C	29.290552537513	-37.143283531240	-62.373023172265
O	29.330125804554	-37.274298631967	-61.146582998770
H	31.100904144389	-37.785939679117	-63.318329645407
O	28.648754452386	-36.231720000832	-63.030878768016
H	29.822602063956	-30.135619702896	-66.196089078303
C	30.555210449864	-30.862260932179	-65.831951342396
H	31.065076319944	-31.289381021151	-66.699086780837
C	29.877961646636	-31.956292697990	-65.004592512762
C	29.162339392591	-31.377583755282	-63.783987904407
C	28.464026242148	-32.390044211117	-62.900295864993
O	28.186429261141	-33.529103399873	-63.391564162953

O	28.176042498276	-32.005868975239	-61.721435088791
H	36.281377094445	-31.594616742739	-63.692786901484
C	36.822737560164	-32.178259430662	-62.934187872619
H	37.552341086022	-32.868361877906	-63.352430641124
C	35.834015563671	-32.937206501635	-62.041609224607
C	35.061145839772	-34.017544040393	-62.804314608004
C	34.026586912373	-34.757389647894	-61.958514473195
N	32.953016315146	-33.836837688225	-61.566332330164
C	31.993649828769	-34.097380152502	-60.672606784664
N	31.913039971657	-35.299604929536	-60.092714305681
N	31.145522067960	-33.134105548509	-60.311968673130
H	20.765532683304	-27.013382950292	-52.914340933106
C	21.484408418101	-26.288277092606	-53.323534289474
C	22.889496886864	-26.458979195224	-52.757946715658
C	23.551267748256	-27.769457043911	-53.152353835071
N	23.637098452544	-27.927131505416	-54.646375654618
Cl	21.807421277001	-30.386615147601	-55.899426757754
O	30.492478649006	-30.725620144746	-58.499694605858
O	25.319496400589	-25.809585544418	-58.009756770234
O	26.485683138855	-39.135880921743	-61.710374959014
O	25.453425832271	-38.805849027203	-56.344667881178
O	23.867840544248	-42.690270974929	-63.252332316520
O	28.687859112159	-32.523876210580	-51.950235791000
O	24.758452534094	-31.657073471651	-54.921224187466
O	28.023894041447	-41.749838311979	-61.895409572948
O	25.985847459178	-33.354968642892	-51.441090030930
O	27.390496870187	-35.545859375136	-54.378207134713
O	24.372519126546	-39.973181962130	-63.352325116275
O	26.064942382377	-44.192083276953	-62.661208268858
O	25.754507652287	-37.545960599368	-53.856220252322
O	26.032683296241	-29.104519192735	-55.081230758023
O	23.366044330023	-25.557436364991	-56.305295605484
H	26.709248670960	-36.182099338948	-54.039798259954
H	28.209421154304	-35.751358548788	-53.876992374648
H	26.847522076923	-32.980389451283	-51.712735393161
H	29.286957704630	-33.069349224239	-51.404249062868
H	25.166667749101	-38.665826751507	-57.283958274876
H	26.172843201276	-39.477372757504	-56.412673503141
H	25.599242571645	-37.971686292666	-54.729401276387
H	24.916177234182	-37.629886581245	-53.411110429528
H	25.307202279190	-32.717736862761	-51.724407615890
H	23.984059195668	-31.426197136401	-55.471405321974
H	24.379269066554	-31.707832618896	-54.009763393378
H	27.196848669915	-41.315371092794	-57.187642490092
H	25.059586258407	-39.729359704400	-62.693508205618
H	26.637244669748	-39.268336215431	-60.756713088149
H	26.454461091626	-38.154842310501	-61.795439961250
H	24.033658264722	-41.718099704182	-63.295474500955
H	23.411516625455	-42.852778549062	-62.397356753321
H	24.723389418009	-39.616776568506	-64.191466899286
H	26.351483707542	-28.839867211378	-55.981461100994
H	25.801237458191	-30.055445916843	-55.117654236195
H	24.568756604486	-28.367232839050	-54.888852335712
H	22.905930107828	-28.560028771202	-55.012138584872
H	23.561462034567	-27.020715600724	-55.152837825721
H	21.588097242785	-31.021255570074	-53.628792677131
H	20.561844862125	-31.234100035891	-52.236392199246

H	29.201082418514	-32.327865841806	-52.752642413780
H	28.739390818435	-41.429894881757	-61.313860990137
H	27.521143426278	-40.943173512368	-62.100228547134
H	29.812566920465	-31.410132019907	-58.694012325241
H	31.918984231477	-31.269044743376	-57.583579211619
H	29.963652572561	-30.029209850624	-58.043597769225
H	26.724611486796	-43.594728167005	-62.272511644244
H	25.323194294065	-43.593777951445	-62.937045106062
H	31.515335956734	-42.709697454866	-58.423402942861
H	30.624249660582	-42.866540842776	-59.901356166390
H	24.189031159397	-25.610546267297	-56.862681290791
H	23.392776951544	-24.693608996892	-55.873799169991
H	24.681373827158	-25.738584167730	-58.763320553115
H	25.802215887747	-26.677157404083	-58.026675322189
H	32.337389219870	-36.098788259109	-60.538013753957
H	31.281219612621	-32.187449575299	-60.631447786813
H	31.148382550435	-35.481802824957	-59.441199220371
H	30.260263325157	-33.340941289325	-59.852465871393
H	32.891093044182	-32.958808530862	-62.062220561552
H	23.494547034128	-33.512275940123	-58.919957133813
H	21.900107905008	-35.239060472037	-62.391649926770
H	20.004312232454	-34.056661022632	-61.188464117367
H	25.207620862556	-32.852637123905	-62.707291345970
H	22.969153026574	-31.526429163838	-62.454433930107
H	26.135612744495	-32.596992269964	-65.050465624556
H	22.567259007705	-30.402856839255	-64.680571894164
H	27.572680133094	-43.456793763758	-59.242137426818
H	25.777179108019	-45.277149921817	-59.097883368016
H	24.901468202233	-42.323927242157	-56.213158624699
H	32.726848100574	-34.174053774748	-55.109744140562
H	21.637647680309	-29.677484186765	-58.226243787532
H	21.829147714607	-42.953189401986	-57.880562523933
H	28.884803855873	-37.246940410432	-64.801091578125
H	27.743275297984	-36.982669578160	-67.082524110896
H	37.382336611796	-31.456088174889	-62.330899533710
H	31.297239527659	-30.320186958631	-65.238220200185
H	30.669541532734	-28.042690394538	-58.072743796543
H	23.365294843574	-36.226995659081	-54.670988979751
H	25.915721376950	-35.795043454565	-66.631850127606
H	27.695619648843	-39.867481193849	-66.625217796558
H	20.762393036684	-36.697613770059	-56.202045669168
H	30.010399475029	-39.109367519174	-62.884327757335
H	19.762171095653	-30.806552506954	-58.314074154331
H	23.005743277332	-28.008938256457	-59.030759283353
H	23.037485209145	-45.259180211677	-59.285967430634
H	32.945434698534	-31.034366254671	-53.784650727194
H	32.678928061740	-42.580830248071	-54.266310396429
H	21.720839699939	-41.229153599424	-60.913979850469
H	29.826730247871	-34.188312860966	-54.595990042900
H	22.614599620614	-39.041636336228	-58.463016677283
H	24.042538720119	-40.666201884106	-60.625699327601
H	23.622699605603	-35.569756656817	-65.576965475397
H	19.743864353757	-33.324828918792	-58.393187009871
H	23.832193416959	-30.726964434234	-58.157285824037
H	24.896145570178	-28.926158690898	-60.395845381989
H	30.128792795361	-36.639221296045	-55.592870115638
H	31.997656290422	-30.105845560528	-55.806074691317

H	28.218613064140	-26.303947579403	-58.700544985732
H	28.615476162449	-38.604278003192	-55.213806830096
H	30.339153744533	-38.994101190047	-53.484042557126
H	30.236392238626	-43.193374941972	-54.341165045724
H	28.533686274823	-42.803707587595	-56.075082014533
H	31.735702316905	-40.489860951103	-52.218403500994
H	23.949093264581	-45.961551832597	-57.064303462841
H	23.757959877063	-37.208504872772	-64.969895878000
H	21.220935872198	-32.550094117371	-57.841428003792
H	25.029308927597	-29.458437987203	-57.999338213609
H	23.959201878741	-30.378814485325	-60.715118532693
H	28.712911903927	-27.710354914915	-59.636068172206
H	22.192365965253	-38.857504221252	-60.166508665964
H	30.622963647436	-32.686898350657	-64.674317159236
H	29.851932462004	-30.813168709236	-63.150569676998
H	29.159756064528	-32.500106282673	-65.623485827783
H	34.491867087255	-35.158395667099	-61.052915816801
H	34.559605018745	-33.578985386836	-63.675704756865
H	36.372937306267	-33.409015627337	-61.212408960790
H	35.760664205557	-34.763994162346	-63.192231158265
H	33.605406674582	-35.592679392732	-62.528397407542
H	33.763049505421	-30.184110448322	-55.947429744813
H	31.720940404793	-40.759586549700	-57.257043542573
H	30.378469566783	-39.634540052159	-57.533426242261
H	26.431716635727	-29.898549436620	-66.932665991173
H	24.666560138979	-31.491756783715	-67.495497642658
H	31.592949267267	-35.967433721603	-56.289794887911
H	23.403707048798	-30.365222783012	-67.030672542728
H	23.243222907205	-44.540283178438	-56.304717853832
H	32.514595011487	-34.705979761276	-52.798450611791
H	31.783072380358	-37.002944044167	-51.188187701235
H	32.851265624743	-35.657999077741	-50.755348133719
H	23.210132438088	-32.229197397682	-49.962393288539
H	21.706363982860	-31.275592339563	-49.966640588141
H	21.664440805426	-34.580688698464	-53.981180661709
H	23.697450747411	-36.816783935233	-57.071089787303
H	21.281049993537	-34.227769708082	-55.671672752381
H	24.121432850288	-35.130482106112	-56.784007187903
H	22.861139744539	-33.785692217217	-55.016992718381
H	22.578892655551	-35.536149000875	-57.540421509987
H	22.999724945241	-28.627804841555	-52.769926454180
H	22.868116077446	-26.413883451570	-51.665253010623
H	21.471100772775	-26.381901281272	-54.412190786406
H	24.570335064310	-27.818058456536	-52.769214636630
H	23.522604991692	-25.630400979903	-53.092840224392
H	21.102522413135	-25.291936460124	-53.084335600938
C	29.261833408720	-35.684476927681	-48.427545171921
H	29.613754897251	-34.817374086901	-47.866393786033
H	30.545791086300	-37.095180363043	-49.376831323827
H	29.057824229876	-36.501907899519	-47.738729901413
H	28.341617375038	-35.413267461923	-48.950326785308
H	31.288456316233	-42.189960585705	-52.156491782607
H	35.134447202242	-32.224757151197	-61.591528731901
H	23.998058148353	-40.960016276738	-58.889922466617
H	28.390317171376	-30.664915065743	-64.100524116698
H	24.138729172952	-49.166056217509	-60.114421651395
N	24.354972471601	-46.510173576140	-61.711340176364

H	23.129208110633	-48.033083002990	-59.214772476715
H	22.780126019579	-48.357419684647	-60.933180604208
H	24.968816247014	-45.741068682448	-61.998962926196
H	23.572171022597	-46.746879859778	-62.300760089468
